# Systemic epigenome-wide association study of elk treponeme-associated hoof disease

**DOI:** 10.1038/s41598-023-42546-8

**Published:** 2023-09-16

**Authors:** Margaret A. Wild, Kyle R. Taylor, Eric E. Nilsson, Daniel Beck, Michael K. Skinner

**Affiliations:** 1 Department of Veterinary Microbiology and Pathology, College of Veterinary Medicine, Washington State University, Pullman, WA 99164 USA; 2 Washington Animal Disease Diagnostic Laboratory, Washington State University, Pullman, WA 99164 USA; 3 Center for Reproductive Biology, School of Biological Sciences, Washington State University, Pullman, WA 99164-4236 USA

**Keywords:** Microbiology, Ecology, Pathogenesis

## Abstract

Treponeme-associated hoof disease (TAHD) is an emerging disease of elk (*Cervus canadensis*) in the U.S. Pacific West. Because environmental epigenetics is the primary molecular process that mediates environmental factor impacts on a host organism and disease, the role of epigenetics in TAHD etiology was examined. The current study was designed to examine potential effects of TAHD on systemic epigenetic modifications in infected elk over a range of TAHD lesion severity. Leg tendons that contain predominantly fibroblast connective tissue cells were used to isolate fibroblast cells for epigenetic analysis in unaffected and TAHD-positive male and female Roosevelt and Rocky Mountain elk. Differential DNA methylation regions (DMRs) between the unaffected and TAHD-positive elk were identified for both female and male elk. The presence of TAHD was associated with alteration of the connective tissue cell epigenetics, and DMR associated genes identified. Therefore, the infected elk were found to have a systemic epigenetic alteration that was associated with the disease, despite pathology being generally limited to feet. If the elk germline epigenetics is altered then generational transmission of susceptibility to TAHD may impact subsequent generations through epigenetic inheritance. This first study of epigenetic changes associated with disease in elk suggests that TAHD promotes a systemic effect on the elk epigenetics which could exert health impacts on the elk.

## Introduction

Treponeme-associated hoof disease (TAHD) is an emerging disease of elk (*Cervus canadensis*) in the U.S. Pacific West. The cause of TAHD is uncertain, but *Treponema* spp., and some other bacteria, are thought to be important components of a polybacterial infection^1–3^. Host and environmental factors that increase susceptibility to disease may also promote the occurrence of TAHD and require further investigation^2,3^.

Clinical signs consistent with TAHD were initially described in elk in southwestern Washington, USA, in winter 2008–2009 during investigation of a marked increase in reports of limping elk^4^. Characteristic hoof lesions include mild skin ulceration in the interdigital space, severe sole ulceration, and deformed, asymmetric, overgrown, or sloughed hooves. These lesions result in lameness often accompanied by debilitation^2^. Sex and age predilection have not been reported. Minor lesions have been detected in elk as young as 3 months of age and severe lesions occur in 9 month old elk^2^. Although TAHD lesions are generally limited to the feet, increased antler asymmetry has been associated with TAHD in male elk^5^.

The present geographic distribution of TAHD includes portions of Washington, Oregon, Idaho, and California^3^. Two ecotypes of elk inhabit this area: Roosevelt elk, which generally occur in western coastal ranges, and Rocky Mountain elk which occur in inland areas^5^. The disease was initially reported in Roosevelt elk with^4^. Reports of TAHD in areas with Rocky Mountain elk tend to be less frequent and more sporadically distributed than in areas where Roosevelt elk occur^3,5^. It is unknown whether these apparent differences are attributable to ecotype, environmental factors, pathogen occurrence and transmission, or other factors.

Epigenetics refers to ‘the molecular factors and processes around the DNA that regulate genome activity independent of DNA sequence, and that are mitotically stable’^6^. Epigenetic processes can be altered by exposure to environmental factors such as nutrition or toxicants, or infectious agents^7,8^. Epigenetic factors include DNA methylation, histone modifications, changes to chromatin structure, and expression of non-coding RNAs^9^. The current study evaluates changes in DNA methylation, which involve methyl group attachment to cytosine residues of DNA that are adjacent to guanine residues (*i.e.,* CpG sites). Epigenetics co-evolved with DNA sequence to provide a molecular mechanism to control gene expression and provide a molecular mechanism for environmental factors to regulate biology. Previously, disease etiology and phenotypic variation have been shown to be mediated through both epigenetic and genetic mechanisms^10^. However, epigenetics is the initial mechanism involved in responding to the environment or infectious disease^11^.

The current study investigates the hypothesis that systemic epigenetic DNA methylation changes are associated with the occurrence of TAHD. Elk leg tendon samples were opportunistically available for study from the lower limbs of elk submitted for TAHD surveillance by collaborating wildlife management agencies^3^. For these tendon samples the primary cell type present is fibroblasts (see Results below). Having predominantly one cell type present in samples is critical when performing epigenetic studies, since the epigenetic states are cell specific for each cell type and are the mechanism that controls cell type specificity and differentiation^12^. In a mixed cell population, if the proportion of cell types changes between samples, then this could erroneously suggest a change in DNA methylation when none had occurred. In the current study DNA methylation is measured by isolating DNA from the tendon samples. The regions of the genome that are methylated are identified using a methylated DNA immunoprecipitation (MeDIP) procedure. The amount of DNA methylation at each region was analyzed using next generation sequencing, as described in Methods. Genomic sites are then identified where DNA methylation levels differ with infection status between different study groups of elk. These epigenetic sites are termed Differential DNA Methylated Regions (DMRs). TAHD-positive elk were compared to unaffected elk for both the Rocky Mountain and Roosevelt elk ecotypes. Samples from male and female elk were analyzed separately, as there are sex-specific epigenetic differences between samples from males and females^13,14^.

The presence of epigenetic effects in the tendon fibroblast would suggest a systemic impact associated with TAHD infection on all cell types in the host. The DMR associated genes are identified to explore the systemic impacts (i.e., all organism cell types) of disease status using the tendon fibroblasts. Observations contribute to understanding of the disease pathology, and future epigenetic testing in elk might be useful as a tool for wildlife disease investigation and management. Epigenetic changes may identify some elk as being more susceptible to TAHD. Alternatively, specific epigenetic changes may be a marker of early infection. Results from these studies may provide the proof of concept that epigenetic changes are associated with TAHD.

## Results

Study samples were obtained from Rocky Mountain elk collected in Washington, Idaho, and South Dakota and from Roosevelt elk from Washington, Oregon, and California. Only cases with required metadata (location of collection and sex) and confirmed diagnosis of TAHD present or not detected were included in the analysis. Treponeme-associated hoof disease was confirmed present (i.e., TAHD-positive) in 21 Roosevelt elk (15 female, 6 male) and 8 Rocky Mountain elk (5 female, 3 male) and not detected (i.e., unaffected) in 24 Roosevelt (14 female, 10 males) and 26 Rocky Mountain elk (14 female, 12 male). Severity of lesions ranged from minor to severe (grade I-IV;^2^) (S1 Table).

Representative tendon samples were fixed and stained (n = 3) and cell nuclei counted to determine the relative proportion of cell types using morphological criteria. Samples were found to have 68% ± 1.7% (mean ± Sd. Dev.) dense connective tissue fibroblasts, 26% ± 4.4% loose connective tissue fibroblasts, and 6% ± 6.2% other cell types, such as vasculature (S1 Fig). Samples were therefore approximately 92% fibroblasts, which is of adequate purity to perform reliable DNA methylation analyses.

DNA was isolated from the samples and methylated DNA evaluated using methylated DNA immunoprecipitation followed by next generation sequencing (MeDIP-seq). Gene sequencing read depth is therefore a measure of the level of DNA methylation at any particular genomic site (see Methods). DNA methylation levels at every region along the genome were compared between TAHD-positive and unaffected groups to determine statistically significant differential DNA methylated regions (DMRs) (see Methods).

**Figure 1:**
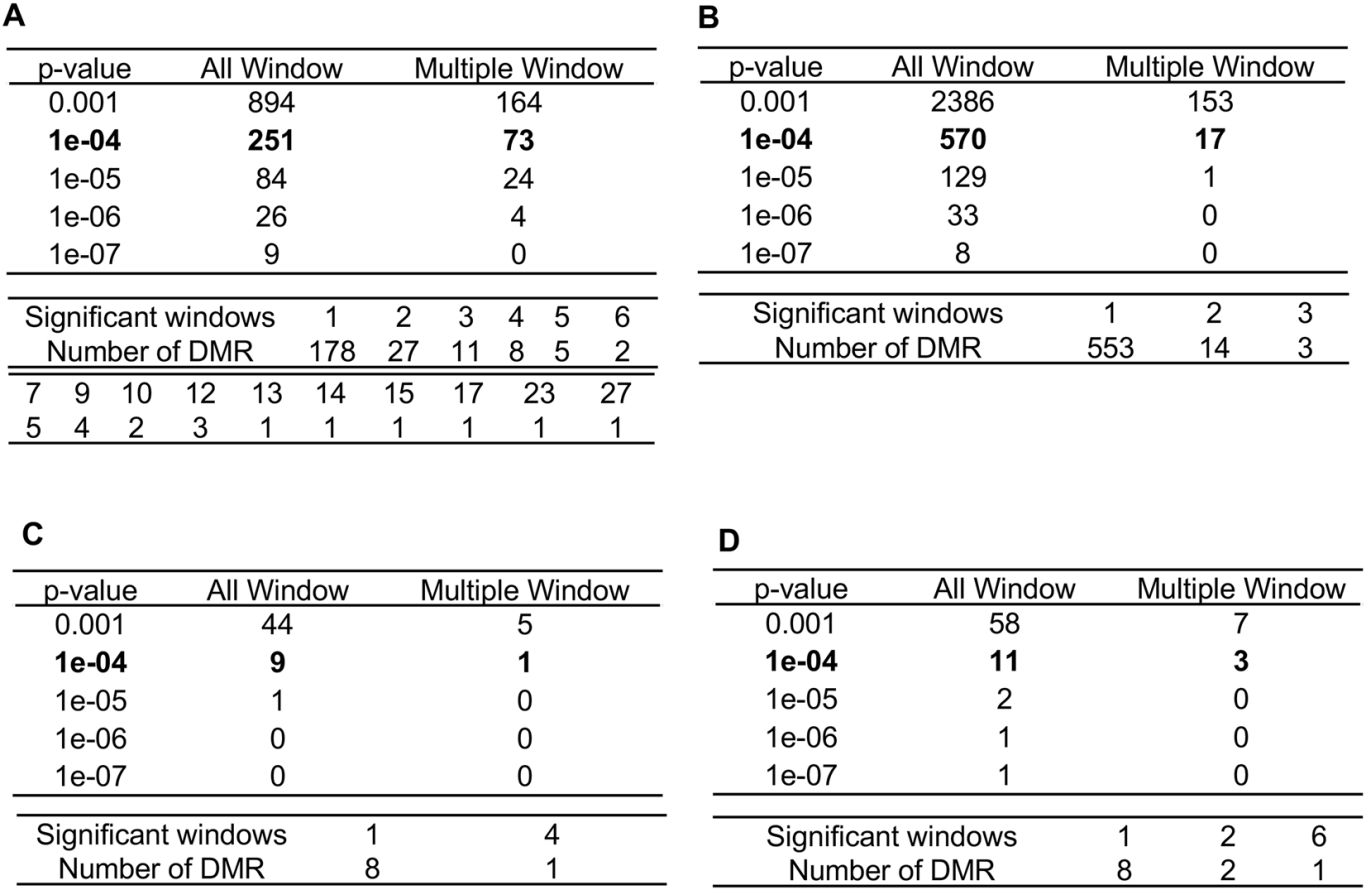
DMR identification and numbers. The number of DMRs found using different p-value cutoff thresholds. The All Window column shows all DMRs. The Multiple Window column shows the number of DMRs containing at least two significant windows (1000 bp each). The number of DMRs with the number of significant windows (1000 bp per window) at a p-value threshold of p < 1e−04 for DMR is presented. (**A**) Rocky Mountain female elk DMRs. (**B**) Roosevelt female elk DMRs. (**C**) Rocky Mountain male elk DMRs. (**D**) Roosevelt male elk DMRs.

The number of DMRs at different statistical thresholds was determined for male and female Rocky Mountain and Roosevelt elk samples (Fig. 1). DMRs at a significance level of p < 1 × 10^−4^ were used for subsequent analyses. For both female comparisons, a p-value < 1 × 10^−4^ resulted in DMRs with a false discovery rate (FDR) < 0.05. The DMR name, chromosomal position, length, statistical significance levels, CpG density, and increase or decrease fold change in DNA methylation are shown for each DMR in S2 Table for Rocky Mountain females, S3 Table for Roosevelt females, S4 Table for Rocky Mountain males, and S5 Table for Roosevelt males. Also shown are any known genes associated with each DMR site, and functional categories for these genes.

**Figure 2:**
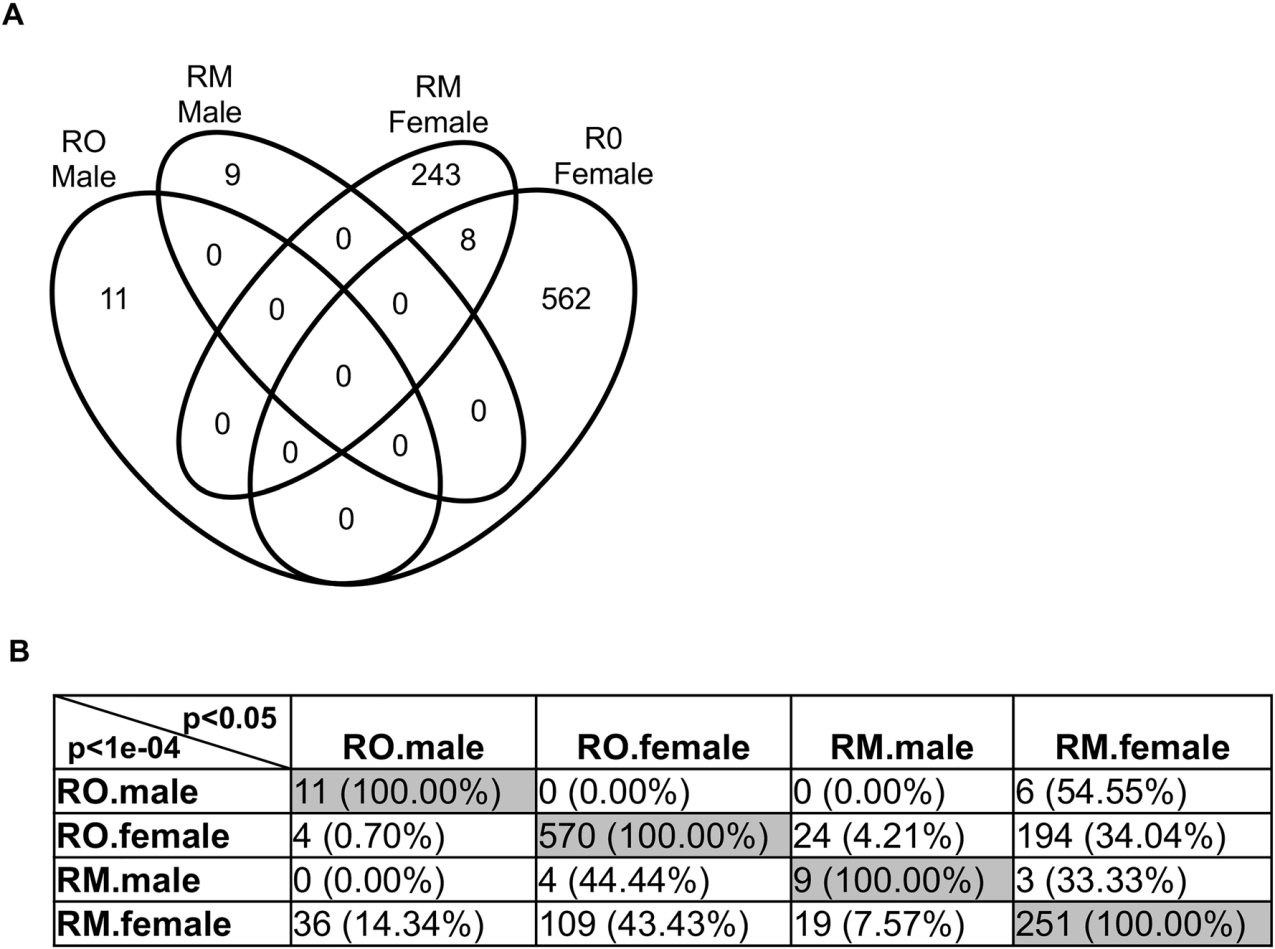
DMR overlap. (**A**) Venn diagram of all DMR overlap (p < 1e−04). (**B**) Extended overlaps (p < 1e−04 vs. p < 0.05). The number of DMRs overlapped and percentage presented.

Very few DMR at p < 1 × 10^−4^ overlapped with each other between Rocky Mountain and Roosevelt males and females (Fig. 2A). However, if one compares the DMRs present in one group at p < 1 × 10^−4^ with the larger number of DMR present in the other groups at a less stringent statistical level of p < 0.05, then it can be seen that Roosevelt female DMRs have a 34% overlap with Rocky Mountain female DMRs (Fig. 2B). Conversely, Rocky Mountain female DMRs at p < 1 × 10^−4^ have a 43% overlap with the Roosevelt female DMRs at p < 0.05.

The male Rocky Mountain and Roosevelt elk sample sets both had very few TAHD-positive animals. This affected the ability of the analysis to detect DMRs, and so relatively few DMRs were identified, compared to Roosevelt and Rocky Mountain females (Fig. 1C,D). These DMRs were characterized for chromosomal position, length, statistical significance levels, CpG density and increased vs. decreased methylation levels (S4, S5 Tables). Little subsequent male DMR analysis was performed, owing to the few DMR identified.

**Figure 3:**
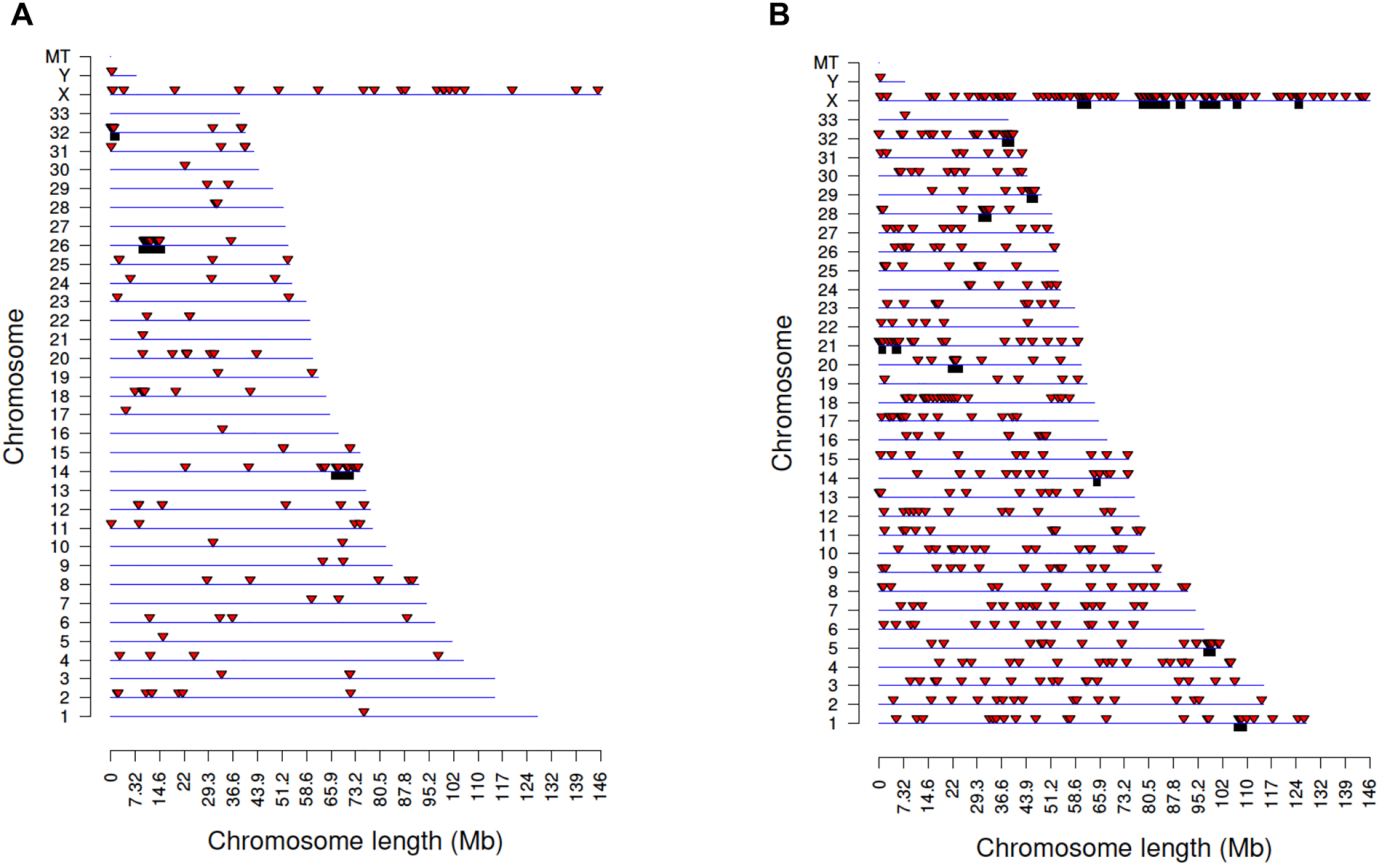
DMR Chromosomal locations. The DMR locations on the individual chromosomes is represented with a red arrowhead and a cluster of DMRs with a black box. All DMRs containing at least one significant window at a p-value threshold of 1e−05 for DMR are shown. (**A**) Rocky Mountain female elk DMR. (**B**) Roosevelt female elk DMR.

The DMRs are localized onto most all chromosomes of the genome for female Rocky Mountain and Roosevelt elk (Fig. 3A,B). The Rocky Mountain elk, having fewer DMRs, had two chromosomes (27 and 33) with no DMRs present. There was limited overlap between the DMRs of the Roosevelt and Rocky Mountain females at p < 1 × 10^−4^ (S6 Table). Two of the eight overlapping DMRs were present on the large X chromosome, and six of eight DMRs were associated with uncharacterized loci (LOC) genomic regions of the genome.

**Figure 4:**
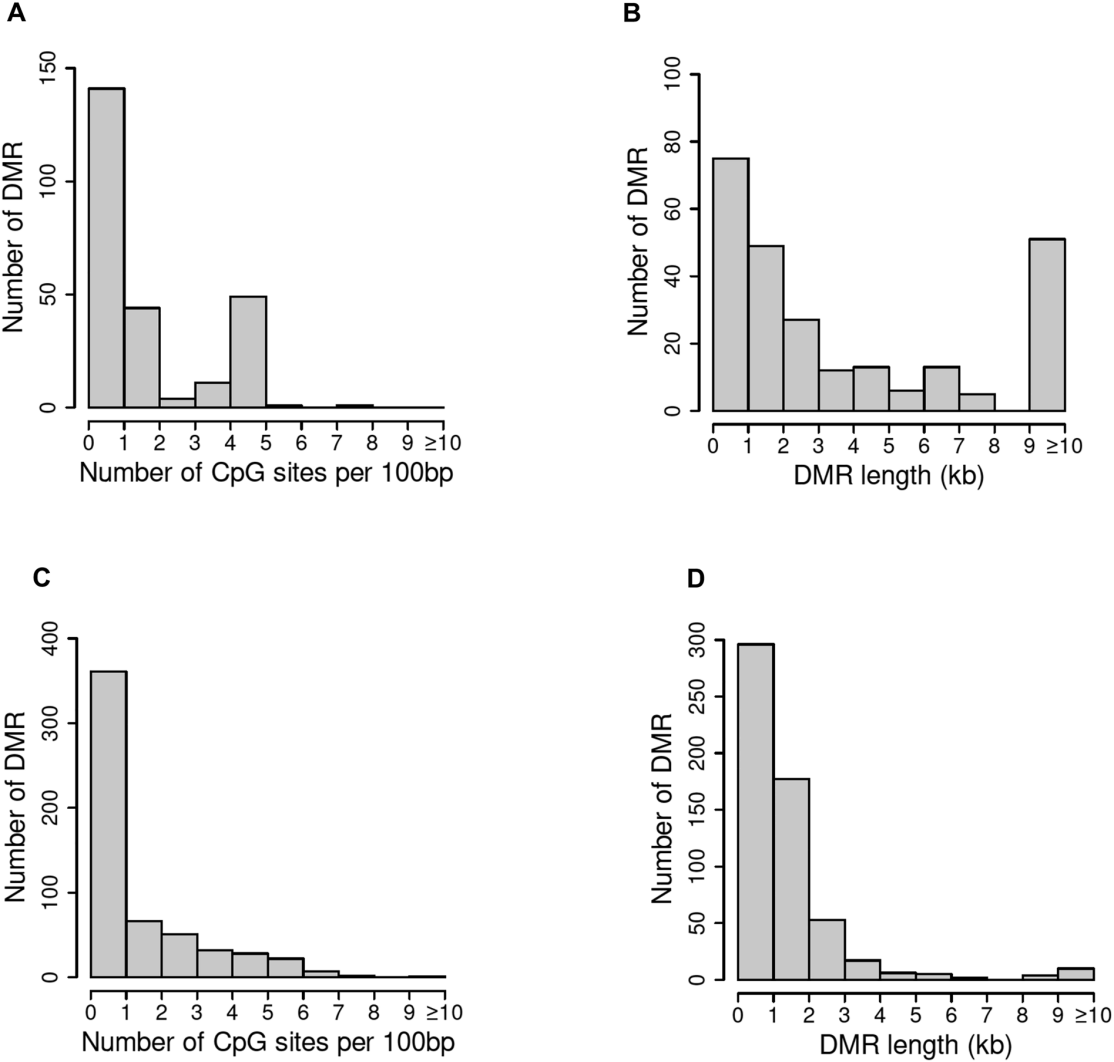
DMR genomic features (CpG density and length). (**A**) Rocky Mountain female elk DMR CpG density. (**B**) Rocky Mountain female elk DMR length. (**C**) Roosevelt female elk DMR CpG density. (**D**) Roosevelt female elk DMR length.

**Figure 5:**
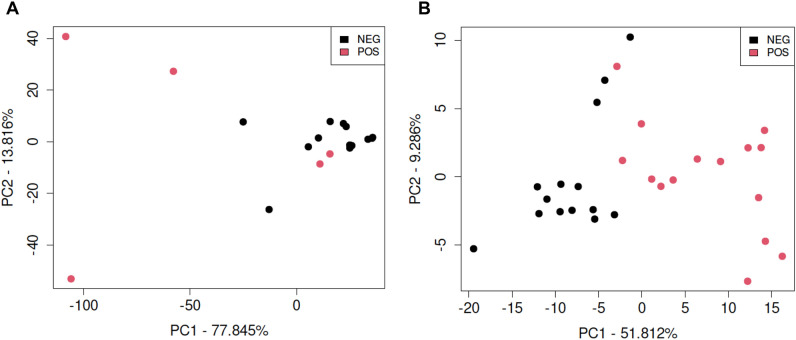
DMR principal component analysis (PCA). The first two principal components are used. The underlying data is the RPKM read depth for DMR genomic windows. (**A**) Rocky Mountain female elk DMR biomarkers. (**B**) Roosevelt female elk DMR biomarkers.

The CpG density (number of methylatable CpG sites per 100 base pairs) of DMRs in female elk is presented in Fig. 4A,C. All the Roosevelt female elk DMRs had a low density of CpG sites (< 5 CpG/100 bp). Interestingly, the Rocky Mountain female elk had predominantly low density CpG DMR, but a reasonable number of high density CpG islands as well, Fig. 4B. The lengths of DMRs were generally between one and three kilobases (Fig. 4B,D). A Principal Components Analysis (PCA) plot of the RPKM read depth data of DMRs are shown in Fig. 5A,B. PCA data indicate that samples separate reasonably well into TAHD-positive and unaffected groups for Roosevelt females. Separation is less clear for the Rocky Mountain females. No separation was apparent based on severity of lesion grade or age class in either ecotype.

**Figure 6:**
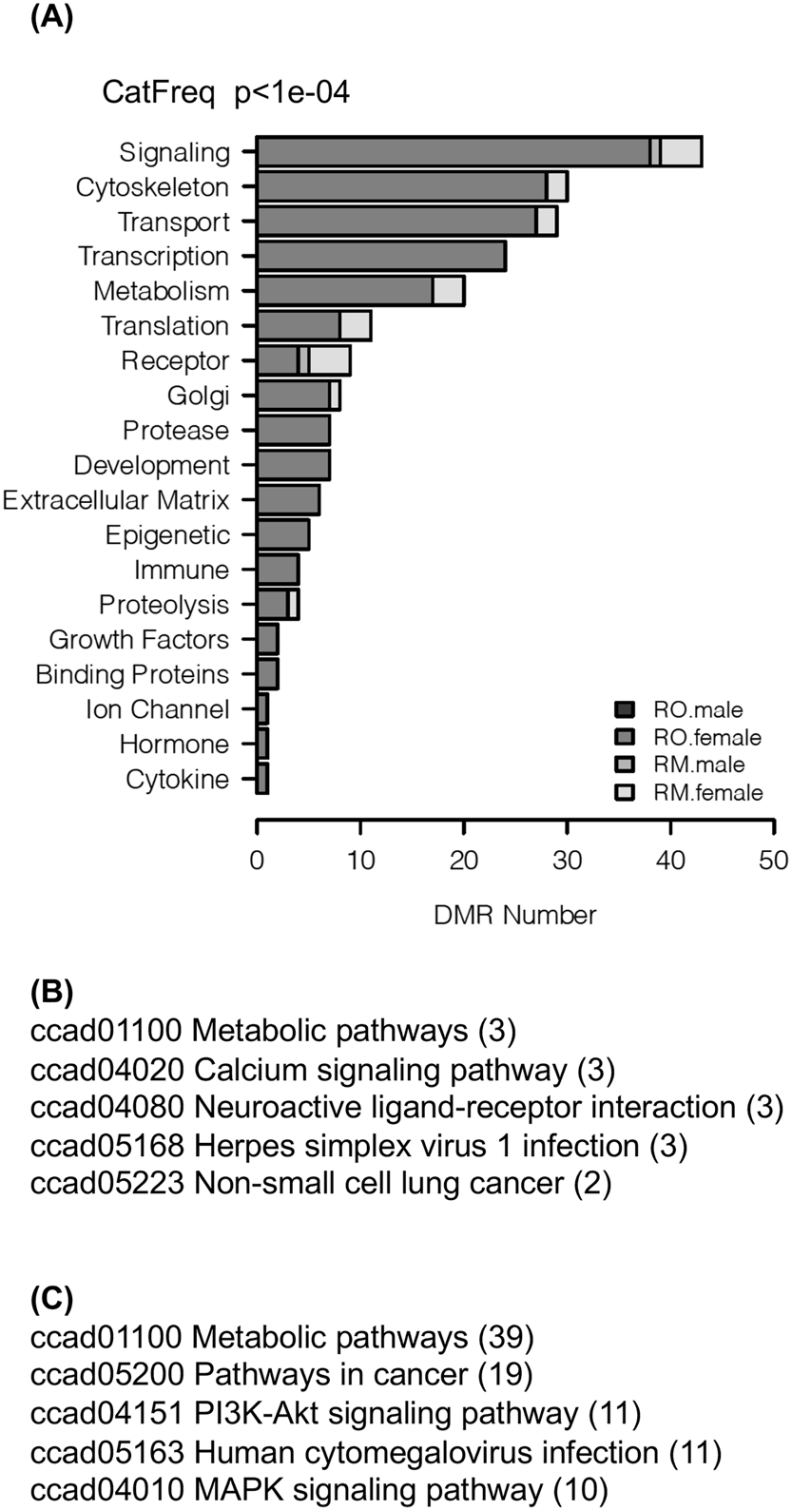
(**A**) DMR gene functional categories (see Methods). (**B**) Rocky Mountain female elk KEGG gene pathway associations. (**C**) Roosevelt female elk KEGG gene pathway associations.

Gene associations with, or near, each DMR for the Rocky Mountain and Roosevelt female groups were identified as described in Methods (S2 and S3 Tables). Forty-nine percent of Rocky Mountain female DMRs were near genes, while 59% of Roosevelt female DMRs were near genes. When known, gene functions were assigned to the DMR-associated genes as shown in S2 and S3 Tables and in Fig. 6. Additional insight into the possible functions of DMR-associated genes was obtained by seeing what physiological pathways the genes participated in using the KEGG database^15^. Metabolic and signaling pathways predominated (Fig. 6B,C).

**Figure 7:**
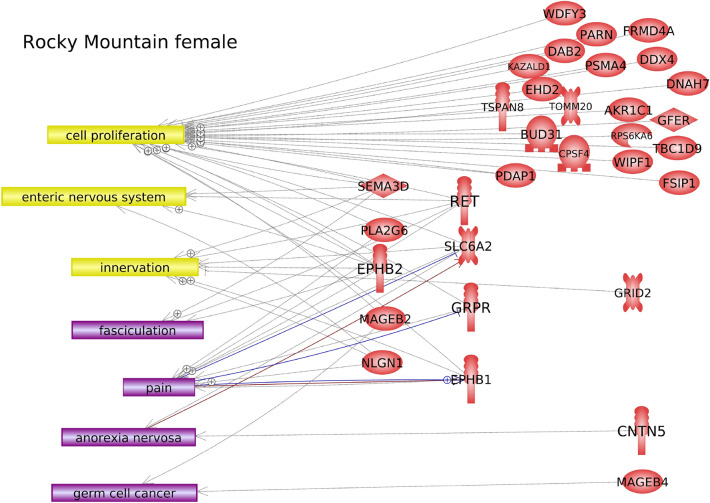
DMR associated gene networks. Rocky Mountain female elk. DMR-associated gene network with related cellular processes (yellow boxes) and diseases (purple boxes) shown, as determined by the Pathway Studio™ database. Gene symbols and correlated names are presented in Supplemental Table S7.

**Figure 8:**
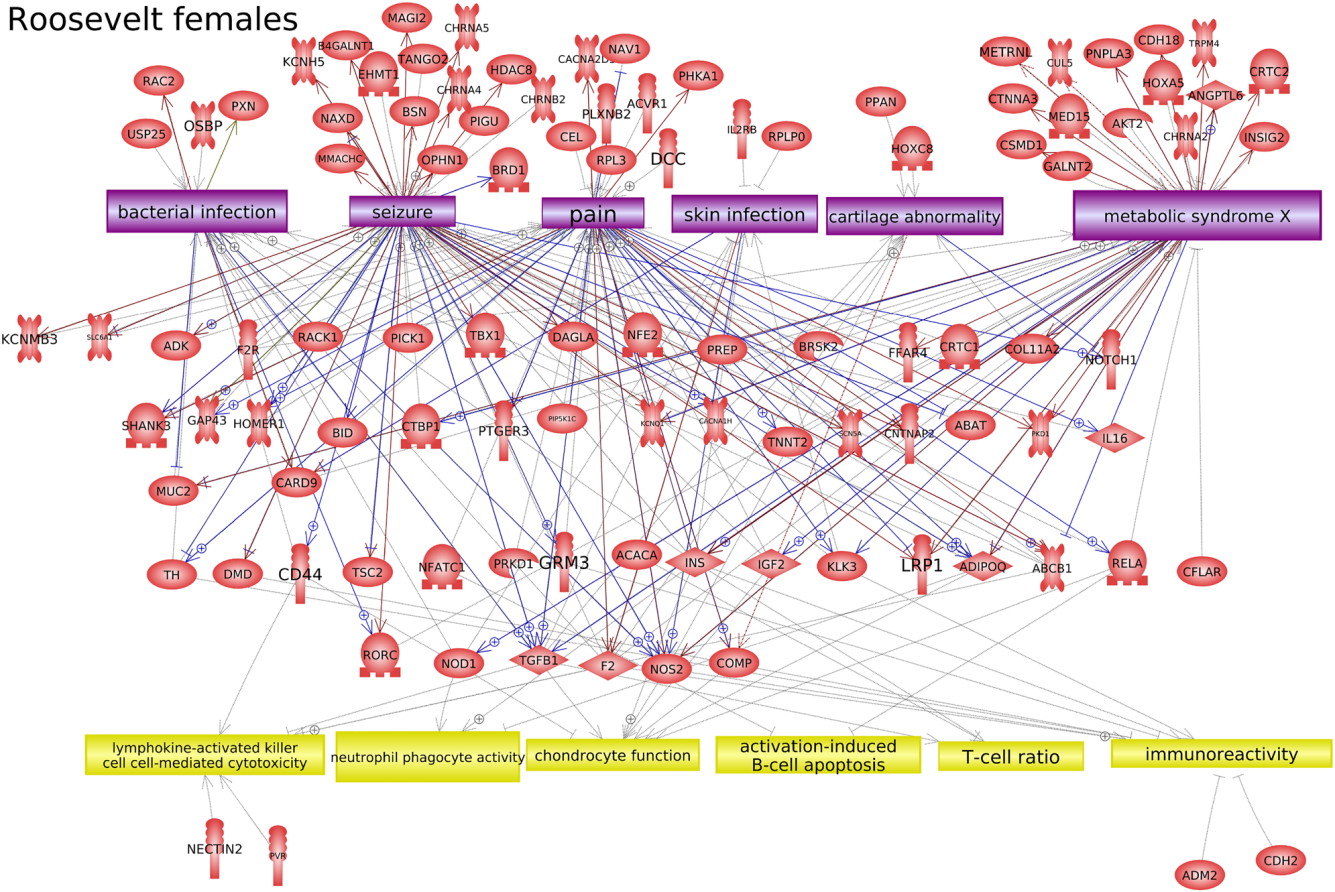
DMR associated gene networks. Roosevelt female elk. DMR-associated gene network with related cellular processes (yellow boxes) and diseases (purple boxes) shown, as determined by the Pathway Studio™ database. Gene symbols and correlated names are presented in Supplemental Table S7.

**Figure 9:**
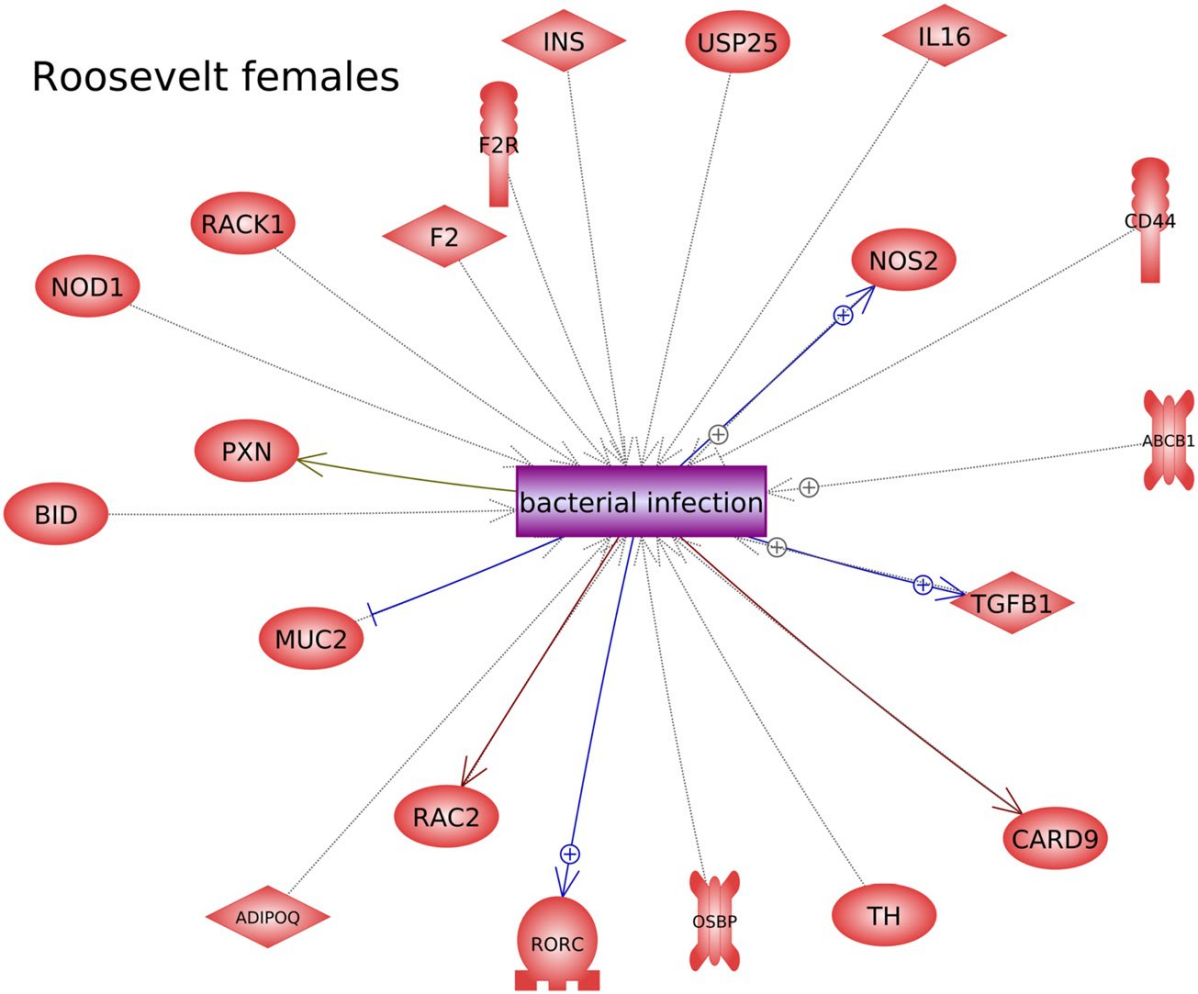
DMR associated genes known to be involved in bacterial infection. Roosevelt female elk. DMR-associated gene network with connections to bacterial infection (purple box) shown, as determined by the Pathway Studio™ database. Gene symbols and correlated names are presented in Supplemental Table S7.

Further investigation into the potential function of DMR-associated genes was performed using Pathway Studio™ software, which identifies connections between genes, cell processes, and diseases in the published literature. For Rocky Mountain female elk, cell processes and diseases that were over-represented in the list of DMR-associated genes included cell proliferation, innervation, and pain (Fig. 7). For Roosevelt female elk, it was found that several genes in proximity to TAHD-related DMRs were over-represented in certain cell processes and diseases, including several immune processes, skin infection, cartilage abnormality, and metabolic syndrome (Fig. 8). In addition, some DMR-associated genes were known to have links to bacterial infection processes (Figs. 8, 9).

## Discussion

Understanding host susceptibility and disease progression is challenging in all species, but particularly in free-ranging wildlife. Diseased wildlife have undocumented health histories so predisposing factors and disease course are generally unknown. Novel approaches, such as epigenetic analysis, may provide insights not otherwise available. Wildlife disease surveillance and sample collection are resource-intensive, with limited availability of samples for study. In this study, samples from 80 elk that were opportunistically collected for disease surveillance provide the first reported epigenetic investigation of disease in elk.

There was a statistically significant epigenetic signature of DMRs in TAHD-positive female Roosevelt and Rocky Mountain elk. That is to say, certain DNA methylation changes occurred relatively frequently in TAHD-positive females compared to unaffected females. This was particularly true in Roosevelt females where a higher number of TAHD-positive animals allowed for a more sensitive analysis and detected a larger number of DMRs. Few DMRs were found to be in common between the Roosevelt and Rocky Mountain females (S6 Table) at a significance level of p < 1 × 10^−4^. It is possible that more overlap could have been detected if more TAHD-positive animals had been present in the Rocky Mountain female group, increasing the sensitivity of the DMR detection. Further studies will be needed to test this hypothesis.

In Roosevelt females with the most robust epigenetic signature, several DMRs were close to genes known to be associated with bacterial infection responses (IL16, CD44, TGFB1, etc. See Fig. 9). This is consistent with the idea that epigenetic changes can occur due to factors such as infection, and these epigenetic changes can then help regulate gene expression to respond to these infections^16^. However, it should be kept in mind that a DMR in proximity to a gene does not necessarily regulate that gene’s expression. It should also be kept in mind that those DMRs not in proximity to a gene may still regulate distant genes through mechanisms such as non-coding RNA expression or changes to chromatin structure^9,17,18^. Much is still unknown about the specific actions of epigenetic changes, especially in species where the genome is not well characterized.

Other studies have shown that epigenetic changes are systemic, such as the ability to detect human susceptibility to female rheumatoid arthritis or preterm birth using buccal cells^19,20^. Analyzed tendon samples were collected proximal to hoof lesions and did not exhibit pathological changes, so observed epigenetic changes likely reflected systemic changes. Full postmortem examinations were not possible for elk used in this study because only lower limbs were submitted. However, although data from complete postmortem examinations of elk with TAHD is limited, other than hoof lesions, no consistent pathologic changes have been reported^2,4^. Therefore, systemic epigenetic changes appear to occur in the absence of observed systemic pathology.

The question arises as to whether the epigenetic changes seen in TAHD-positive elk are a cause of or an effect of infection. That is to say, are elk with certain systemic patterns of DNA methylation more susceptible to infection leading to TAHD? Or, does the presence of TAHD lead to systemic changes in DNA methylation that are reflected in the tendon samples analyzed? In either case, the presence of a TAHD-associated epigenetic signature might be useful. If an epigenetic signature indicates susceptibility to developing TAHD, then detection of this signature may be used as a conservation tool to investigate risk factors that may have led to this increased susceptibility or to focus mitigation efforts on certain populations. Conversely, if specific changes in DNA methylation are a response to infection, then detection of this signature could perhaps be used to identify individuals in the early stages of disease. Epigenetic changes in response to disease may also explain other TAHD-associated changes, such as asymmetrical antlers. DNA methylation patterns are unique in regenerative antler tissue^21^ and systemic epigenetic alternations might influence antler growth. Since the abnormal antler growth is associated with TAHD, the systemic epigenetic impacts could explain the molecular mechanisms involved.

Exposure to environmental factors can lead to heritable changes in epigenetics^9^. Germ line mediated epigenetic transgenerational inheritance has been observed in many species, from rats to plants to *Caenorhabditis elegans* roundworms^22–24^. Therefore, it is possible that epigenetically inherited DNA methylation changes are pre-disposing some elk to acquiring TAHD. Future studies of parents and offspring, combining pathology and epigenetic analyses, will be needed to investigate this possibility.

The results of the current study provide the proof of concept that systemic epigenetic changes are associated with TAHD in elk. In order for epigenetic analysis to become a usable tool in conservation biology, a less invasive sample than a leg tendon would have to be used. Epithelial cells from buccal swabs, or perhaps fecal samples depleted of bacterial DNA, are possibilities for further studies in live elk. The fact that tendon samples showed epigenetic changes in the presence of TAHD, and that the tendon at that position was not a part of the hoof where TAHD pathological changes were present, indicates that these epigenetic changes are systemic and should be detectable in a non-invasive sample. It is recommended that further studies use non-invasive samples having larger sample sizes with more samples from TAHD-positive animals. The goal will be discovering robust epigenetic signatures in both males and females across ecotypes. Future investigations might be expanded to also perform prospective studies to see if healthy elk with a TAHD-associated epigenetic signature are at higher risk of developing TAHD.

In conclusion, epigenetic changes in the form of DMRs were found to be associated with TAHD, especially in female Roosevelt elk. In the future, epigenetic analysis of non-invasive samples could be a valuable tool for investigating and managing TAHD in elk populations.

## Methods

### Sample collection

Lower limbs from TAHD-suspect and apparently normal elk and associated metadata were solicited from state, federal, and tribal collaborators for TAHD surveillance conducted during 2018–2020 as described by Wild et al.^3^. Elk were harvested, culled for management purposes, or found recently deceased. As such, the study was exempt from review by an Institutional Animal Care and Use Committee. Ecotype was inferred from location of collection. Age class of elk was assigned as adult (> 2 years old) or juvenile (< 2 years old).

Frozen lower limbs (−20) were submitted to the Washington Disease Diagnostic Laboratory (WADDL) for TAHD diagnosis and sample collection. Feet were thawed, grossly examined, and sampled under the direction of a board-certified veterinary pathologist. Tissue samples used to determine TAHD status were placed in formalin and processed for histologic examination with H&E and Warthin-Starry silver stains^3^. Cases that exhibited characteristic gross lesions with inflammation and spirochetes on histologic examination were diagnosed as TAHD positive^2^. An approximately 1 cm section of the superficial or deep flexor tendon at about the level of the fetlock was collected from one foot from each elk for epigenetic analysis. Tendon appeared grossly normal, S1 Fig, and was collected proximal to the location of TAHD lesions. The sample was placed in AllProtect Tissue Reagent™ (Qiagen, Germantown, Maryland) storage solution and frozen at −80 until use.

### DNA preparation

Frozen elk tendon samples were thawed for analysis. Genomic DNA from tendon samples was prepared as follows: DNA was isolated from tendon samples by cutting a 2 mm $\times$ 2 mm $\times$ 2 mm cube of tendon and rinsing twice in 1 ml PBS followed by centrifugation at 13,000 g for 1 min to remove the Allprotect Tissue Reagent™. Each sample was minced and incubated with 1 mg/ml collagenase I in 1 ml PBS at 37C with rotation overnight. Samples were then processed using the DNeasy Blood and Tissue Kit™ (Qiagen) to extract DNA, following the manufacturer’s protocols. DNA concentration was measured using the Nanodrop (Thermo Fisher, Waltham, MA).

### Methylated DNA Immunoprecipitation (MeDIP)

Methylated DNA Immunoprecipitation (MeDIP) with genomic DNA was performed as follows: individual DNA samples (2–4 ug of total DNA) were diluted to 130 μl with 1 × Tris–EDTA (TE, 10 mM Tris, 1 mM EDTA) and sonicated with the Covaris M220 using the 300 bp setting. Fragment size was verified on a 2% E-gel agarose gel. The sonicated DNA was transferred from the Covaris tube to a 1.7 ml microfuge tube, and the volume was measured. The sonicated DNA was then diluted with TE buffer (10 mM Tris HCl, pH7.5; 1 mM EDTA) to 400 μl, heat-denatured for 10 min at 95 °C, then immediately cooled on ice for 10 min. Then 100 μl of 5X IP buffer and 5 μg of antibody (monoclonal mouse anti 5-methyl cytidine; Diagenode #C15200006) were added to the denatured sonicated DNA. The DNA-antibody mixture was incubated overnight on a rotator at 4 °C. The following day magnetic beads (Dynabeads M-280 Sheep anti-Mouse IgG; 11201D) were pre-washed as follows: beads were resuspended in the vial, then the appropriate volume (50 μl per sample) was transferred to a microfuge tube. The same volume of Washing Buffer (at least 1 mL 1XPBS with 0.1% BSA and 2 mM EDTA) was added and the bead sample was resuspended. The tube was then placed into a magnetic rack for 1–2 min and the supernatant was discarded. The tube was removed from the magnetic rack and the beads were washed once. The washed beads were resuspended in the same volume of 1xIP buffer (50 mM sodium phosphate ph7.0, 700 mM NaCl, 0.25% TritonX-100) as the initial volume of beads. 50 μl of beads were added to the 500 μl of DNA-antibody mixture from the overnight incubation, then incubated for 2 h on a rotator at 4 °C. After the incubation, the bead-antibody-DNA complex was washed three times with 1X IP buffer as follows: the tube was placed into a magnetic rack for 1–2 min and the supernatant was discarded, then the magnetic bead antibody pellet was washed with 1xIP buffer 3 times. The washed bead antibody DNA pellet was then resuspended in 250 μl digestion buffer with 3.5 μl Proteinase K (20 mg/ml). The sample was incubated for 2–3 h on a rotator at 55 °C, then 250 μl of buffered Phenol–Chloroform-Isoamyl alcohol solution was added to the sample, and the tube was vortexed for 30 s and then centrifuged at 14,000 rpm for 5 min at room temperature. The aqueous supernatant was carefully removed and transferred to a fresh microfuge tube. Then 250 μl chloroform were added to the supernatant from the previous step, vortexed for 30 s and centrifuged at 14,000 rpm for 5 min at room temperature. The aqueous supernatant was removed and transferred to a fresh microfuge tube. To the supernatant 2 μl of glycoblue (20 mg/ml), 20 μl of 5 M NaCl, and 500 μl ethanol were added and mixed well, then precipitated in −20 °C freezer for 1 h to overnight. The precipitate was centrifuged at 14,000 rpm for 20 min at 4 °C and the supernatant was removed, while not disturbing the pellet. The pellet was washed with 500 μl cold 70% ethanol in −20 °C freezer for 15 min then centrifuged again at 14,000 rpm for 5 min at 4 °C and the supernatant was discarded. The tube was spun again briefly to collect residual ethanol to the bottom of the tube and as much liquid as possible was removed with gel loading tip. The pellet was air-dried at room temperature until it looked dry (about 5 min) then resuspended in 20 μl H2O or TE. DNA concentration was measured in Qubit (Life Technologies) with ssDNA kit (Molecular Probes Q10212).

### MeDIP-seq analysis

The MeDIP DNA samples (50 ng of each) were used to create libraries for next generation sequencing (NGS) using the NEBNext Ultra 11 RNA Library Prep Kit for Illumina (San Diego, CA) starting at step 1.4 of the manufacturer’s protocol to generate double stranded DNA. After this step the manufacturer’s protocol was followed. Each sample received a separate index primer. NGS was performed at Washington State University Spokane Genomics Core using the Illumina HiSeq 2500 with a PE50 application, with a read size of approximately 50 bp and approximately 13–34 million reads per sample, and 10–11 sample libraries each were run in one lane.

### Molecular bioinformatics and statistics

Basic read quality was verified using information produced by the FastQC program (FastQC: a quality control tool for high throughput sequence data. Available online at: http://www.bioinformatics.babraham.ac.uk/projects/fastqc). Reads were filtered and trimmed to remove low quality base pairs using Trimmomatic^25^. The reads for each sample were mapped to the GCF 019320065.1 elk genome^26^ using Bowtie2^27^ with default parameter options. The mapped read files were then converted to sorted BAM files using SAMtools^28^. To identify DMR, the reference genome was broken into 1000 bp windows. The MEDIPS R package^29^ was used to calculate differential coverage between control and exposure sample groups. The edgeR p-value^30^ was used to determine the relative difference between the two groups for each genomic window. Windows with an edgeR p-value less than 10^−4^ were considered DMRs. The DMR edges were extended until no genomic window with an edgeR p-value less than 0.1 remained within 1000 bp of the DMR. CpG density and other information was then calculated for the DMR based on the reference genome. DMR were annotated using the NCBI provided annotations. The genes that overlapped with DMR were then input into the KEGG pathway search^15,31^ to identify associated pathways. The DMR associated genes were then sorted into functional groups by reducing Panther^32^ protein classifications into more general categories. A Pathway Studio™ (Elsevier, Inc.) database and network tool was used to assess physiological and disease process gene correlations. All MeDIP-Seq genomic data obtained in the current study have been deposited in the NCBI public GEO database (GEO #: GSE240728).

### Ethical approval

The tissues were provided by state, tribal, and federal wildlife agencies from normal wildlife management agency activities, and submitted to Washington State University for diagnostic testing. Therefore, no live animal activities were involved in the study, but only tissue use and analysis. The Animal Care and Use Committee found this as exempt from any review and determined as exempt status. All methods were carried out in accordance with relevant guidelines and regulations. All methods are reported in accordance with ARRIVE guidelines.

### Supplementary Information

Supplementary Information.

## Data Availability

All molecular data has been deposited into the public database at NCBI https://www.ncbi.nlm.nih.gov/geo/ (GEO # GSE240728) and R code computational tools are available at GitHub (https://github.com/skinnerlab/MeDIP-seq) and https://skinner.wsu.edu/genomic-data-and-r-code-files/. All computational tools can be uploaded for independent use.

## Abbreviations

TAHD: Treponeme-associated hoof disease
DMRs: Differential DNA methylation regions
MeDIP: Methylated DNA immunoprecipitation
PCA: Principal components analysis
