# Supplementary material for: Systemic epigenome-wide association study of elk treponeme-associated hoof disease
Source: Sci Rep. 2023 Sep 16;13:15378. doi: 10.1038/s41598-023-42546-8 (PMC10505176; doi:10.1038/s41598-023-42546-8)
Supplement: Supplementary file 1 — Supplementary Information. [file 41598_2023_42546_MOESM1_ESM.pdf]

Supplementary Figure Legends

Supplemental Figure S1. Elk Tendon Histology. Portions of tendon samples submitted for epigenetic analysis were transferred from AllProtect Tissue Reagent™ to Bouin's solution to fix overnight, then washed three times in 70% ethanol, then sectioned and hematoxylin and eosin stained using standard protocols. **(A)** Microscopic image captured using 10x objective lens. **(B)** Enlarged area bounded by black box in A. Light areas are artifacts of shattering of the tissue section during sectioning.

Supplemental Table S1. Sample Information. Columns indicate: State = State of origin of the sample (WA = Washington; SD = South Dakota; ID = Idaho; OR = Oregon; CA = California). Ecotype ROMO = Rocky Mountain elk; ROOS = Roosevelt elk
Sex F = Female; M = Male. Age A = Adult (≥2 years old); J = Juvenile (<2 years old). TAHD = Histologically confirmed diagnosis of treponeme-associated hoof disease, NEG = TAHD not detected; POS = TAHD positive. Grade = Hoof lesion grade based on a scoring system previously reported [2]. Higher scores represent increasing severity of lesions, e.g., 0 = TAHD lesions not present and 4 = sloughed hoof capsule.

Supplemental Table S2. Rocky Mountain Female Elk DMR p<1e-04. DMR name, chromosome number, start nucleotide site, length (bp), p-value, maximum log fold change (LFC), CpG number and density, gene annotation, and gene category.

Supplemental Table S3. Roosevelt Female Elk DMR p<1e-04. DMR name, chromosome number, start nucleotide site, length (bp), p-value, maximum log fold change (LFC), CpG number and density, gene annotation, and gene category.

Supplemental Table S4. Rocky Mountain Male Elk DMR p<1e-04. DMR name, chromosome number, start nucleotide site, length (bp), p-value, maximum log fold change (LFC), CpG number and density, gene annotation, and gene category.

Supplemental Table S5. Roosevelt Male Elk DMR Table p<1e-04. DMR name, chromosome number, start nucleotide site, length (bp), p-value, maximum log fold change (LFC), CpG number and density, gene annotation, and gene category.

Supplemental Table S6. Overlaps between Rocky Mountain and Roosevelt Female Elk DMRs at p<1e-04. DMR name, chromosome number, start nucleotide site, length (bp), p-value, maximum log fold change (LFC), CpG number and density, gene annotation, and gene category.

Supplemental Table S7. DMR Associated Genes and Names. Gene symbol and name listed.

Supplemental Figure S1

Elk Tendon Histology

A

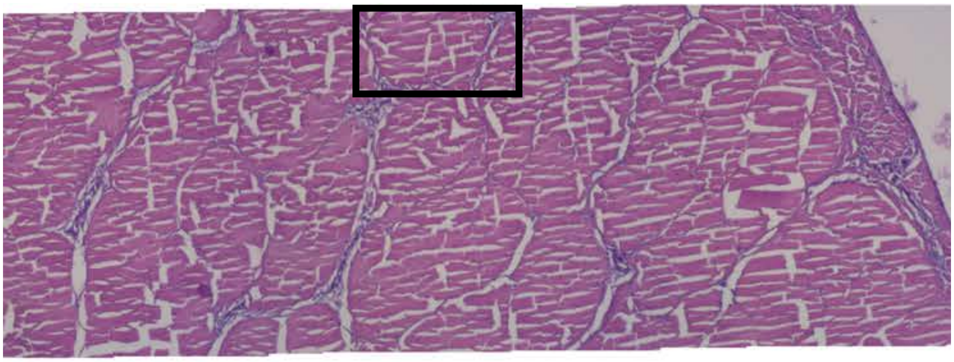

B

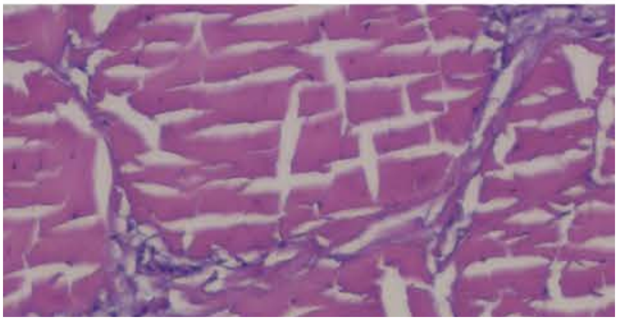

Supplemental Figure S1. Elk Tendon Histology. Portions of tendon samples submitted for epigenetic analysis were transferred from AllProtect Tissue Reagent™ to Bouin's solution to fix overnight, then washed three times in 70% ethanol, then sectioned and hematoxylin and eosin stained using standard protocols. **(A)** Microscopic image captured using 10x objective lens. **(B)** Enlarged area bounded by black box in A. Light areas are artifacts of shattering of the tissue section during sectioning.

Supplemental Table S1
Elk Combined Metadata with Grades

Lid	Sid	Ssid	State	Ecotype	Sex	Age	Tahd	Grade
ET51	RM-F1	WA-066	WA	ROMO	F	A	NEG	0
ET52	RM-F2	WA-105	WA	ROMO	F	A	NEG	0
ET53	RM-F3	WA-069	WA	ROMO	F	A	NEG	0
ET54	RM-F4	SD-08	SD	ROMO	F	A	NEG	0
ET55	RM-F5	WA20-H6	WA	ROMO	F	A	NEG	0
ET56	RM-F6	SD-04	SD	ROMO	F	A	NEG	0
ET57	RM-F7	SD-05	SD	ROMO	F	A	NEG	0
ET58	RM-F8	SD-13	SD	ROMO	F	A	NEG	0
ET59	RM-F9	SD-14	SD	ROMO	F	A	NEG	0
ET60	RM-F10	SD-15	SD	ROMO	F	A	NEG	0
ET61	RM-F11	SD-16	SD	ROMO	F	A	NEG	0
ET62	RM-F12	SD-17	SD	ROMO	F	A	NEG	0
ET63	RM-F13	SD-19	SD	ROMO	F	A	NEG	0
ET64	RM-F14	SD-18	SD	ROMO	F	J	NEG	0
ET65	RM-F15	ID-01	ID	ROMO	F	A	POS	3
ET66	RM-F16	ID-15	ID	ROMO	F	A	POS	4
ET67	RM-F17	WA20-CM50	WA	ROMO	F	A	POS	4
ET68	RM-F18	ID-13	ID	ROMO	F	A	POS	2
ET69	RM-F19	ID-10	ID	ROMO	F	J	POS	1
ET71	RM-M1	ID-018	ID	ROMO	M	A	NEG	0
ET73	RM-M3	WA-067	WA	ROMO	M	A	NEG	0
ET75	RM-M6	SD-06	SD	ROMO	M	A	NEG	0
ET76	RM-M7	SD-02	SD	ROMO	M	A	NEG	0
ET77	RM-M8	ID-025	ID	ROMO	M	A	NEG	0
ET78	RM-M9	ID-026	ID	ROMO	M	A	NEG	0
ET79	RM-M10	SD-12	SD	ROMO	M	A	NEG	0
ET80	RM-M11	SD-10	SD	ROMO	M	A	NEG	0
ET81	RM-M12	SD-09	SD	ROMO	M	A	NEG	0
ET82	RM-M13	SD-11	SD	ROMO	M	A	NEG	0
ET83	RM-M14	WA20-H5	WA	ROMO	M	J	NEG	0
ET84	RM-M16	ID-06	ID	ROMO	M	A	POS	3
ET85	RM-M17	ID-11	ID	ROMO	M	A	POS	2
ET86	RM-M18	ID-08	ID	ROMO	M	A	POS	3
ET87	RM-M19	WA20-CM54	WA	ROMO	M	J	NEG	0
ET1	RO-F1	OR-029	OR	ROOS	F	A	NEG	0
ET2	RO-F2	CA2020-160	CA	ROOS	F	A	NEG	0
ET3	RO-F3	CA2020-168	CA	ROOS	F	A	NEG	0
ET4	RO-F4	CA2020-173	CA	ROOS	F	A	NEG	0
ET5	RO-F5	CA2020-002	CA	ROOS	F	A	NEG	0
ET7	RO-F7	WA18-1808	WA	ROOS	F	U	NEG	0
ET8	RO-F8	WA18-1811	WA	ROOS	F	U	NEG	0
ET9	RO-F10	WA18-1844	WA	ROOS	F	U	NEG	0

ET10	RO-F12	WA18-1847	WA	ROOS	F	U	NEG	0
ET11	RO-F13	WA18-1855	WA	ROOS	F	U	NEG	0
ET12	RO-F14	WA18-1862	WA	ROOS	F	U	NEG	0
ET13	RO-F15	WA18-1863	WA	ROOS	F	U	NEG	0
ET14	RO-F16	CA-04	CA	ROOS	F	A	POS	4
ET15	RO-F17	CA-05	CA	ROOS	F	A	POS	1
ET16	RO-F18	OR-023	OR	ROOS	F	A	POS	1
ET17	RO-F19	OR-024	OR	ROOS	F	A	POS	4
ET18	RO-F20	OR-025	OR	ROOS	F	A	POS	3
ET19	RO-F21	OR-026	OR	ROOS	F	A	POS	2
ET20	RO-F22	OR-028	OR	ROOS	F	A	POS	3
ET21	RO-F23	WA20-CM102	WA	ROOS	F	A	POS	4
ET22	RO-F24	WA20-CM7	WA	ROOS	F	A	POS	3
ET24	RO-F26	WA20-CM110	WA	ROOS	F	A	POS	3
ET25	RO-F27	WA20-CM48	WA	ROOS	F	A	POS	2
ET26	RO-F28	WA20-CM109	WA	ROOS	F	J	POS	3
ET27	RO-F29	CA-02	CA	ROOS	F	U	POS	3
ET28	RO-F30	WA18-1806	WA	ROOS	F	U	NEG	0
ET29	RO-F31	WA18-1812	WA	ROOS	F	U	POS	4
ET30	RO-F32	WA18-1838	WA	ROOS	F	U	NEG	0
ET33	RO-F35	WA20-CM113	WA	ROOS	F	J	POS	2
ET34	RO-M1	CA-06	CA	ROOS	M	A	NEG	0
ET35	RO-M2	WA-124	WA	ROOS	M	A	NEG	0
ET36	RO-M3	CA-11	CA	ROOS	M	A	NEG	0
ET37	RO-M4	CA-12	CA	ROOS	M	A	NEG	0
ET38	RO-M5	CA-13	CA	ROOS	M	A	NEG	0
ET39	RO-M6	CA-14	CA	ROOS	M	A	NEG	0
ET40	RO-M7	CA-15	CA	ROOS	M	A	NEG	0
ET41	RO-M8	WA20-CM105	WA	ROOS	M	A	POS	3
ET42	RO-M9	WA20-CM49	WA	ROOS	M	A	POS	4
ET43	RO-M10	WA20-CM111	WA	ROOS	M	A	NEG	0
ET44	RO-M11	WA20-CM104	WA	ROOS	M	A	POS	4
ET45	RO-M12	CA-01	CA	ROOS	M	U	POS	4
ET46	RO-M13	WA18-1801	WA	ROOS	M	U	POS	3
ET47	RO-M14	WA18-1814	WA	ROOS	M	U	NEG	0
ET48	RO-M15	WA18-1815	WA	ROOS	M	U	POS	2
ET49	RO-M16	WA18-1820	WA	ROOS	M	U	NEG	0

Supplemental Table S1. Sample Information. Columns indicate: State = State of origin of the sample (WA = Washington; SD = South Dakota; ID = Idaho; OR = Oregon; CA = California). Ecotype ROMO = Rocky Mountain elk; ROOS = Roosevelt elk Sex F = Female; M = Male. Age A = Adult (≥2 years old); J = Juvenile (<2 years old). TAHD = Histologically confirmed diagnosis of treponeme-associated hoof disease, NEG = TAHD not detected; POS = TAHD positive. Grade = Hoof lesion grade based on a scoring system previously reported (2). Higher scores represent increasing severity of lesions, e.g., 0 = TAHD lesions not present and 4 = sloughed hoof capsule.

Supplemental Table S2
Rocky Mountain Female DMR p<1e-04

DMR Name	Chr	Start	Stop	Length	# Sig Win	minP	minFDR	maxLFC	CpG #/ 1Kb	CpG Density/ 100 bp	Gene Annotation	Gene Category
1:75699001	1	75699001	75701000	2000	1	4.96E-05	1.98E-02	-0.974	12	0.6	TBC1D9	Signaling
2:1978001	2	1978001	1981000	3000	1	3.46E-05	1.72E-02	-1.568	7	0.23		
2:2306001	2	2306001	2307000	1000	1	2.72E-05	1.65E-02	-1.478	3	0.3		
2:10628001	2	10628001	10630000	2000	1	1.17E-05	1.09E-02	-1.563	20	1		
2:12258001	2	12258001	12259000	1000	1	6.45E-05	2.26E-02	-1.206	3	0.3	LOC122426735	
2:12350001	2	12350001	12355000	5000	2	1.47E-05	1.24E-02	-1.739	34	0.68		
2:20336001	2	20336001	20337000	1000	1	4.23E-05	1.87E-02	-1.206	3	0.3		
2:21542001	2	21542001	21547000	5000	1	5.69E-05	2.13E-02	-1.391	53	1.06	LOC122428517;LOC122437533	
2:71723001	2	71723001	71724000	1000	1	6.99E-05	2.35E-02	-1.073	7	0.7		
3:33172001	3	33172001	33174000	2000	1	6.85E-05	2.33E-02	-1.245	6	0.3	SEMA3D;LOC122438241	Signaling
3:71451001	3	71451001	71453000	2000	1	1.82E-05	1.38E-02	-1.242	5	0.25		
3:71540001	3	71540001	71541000	1000	1	9.92E-05	2.74E-02	-1.001	7	0.7		
4:2739001	4	2739001	2740000	1000	1	1.76E-08	6.68E-04	1.758	32	3.2	MAN2A1;LOC122439413	
4:11857001	4	11857001	11858000	1000	1	7.68E-06	9.48E-03	-1.393	1	0.1		
4:24930001	4	24930001	24931000	1000	1	5.43E-05	2.07E-02	-0.904	5	0.5		
4:97892001	4	97892001	97893000	1000	1	2.35E-05	1.54E-02	-1.19	2	0.2		
5:15653001	5	15653001	15661000	8000	5	3.21E-07	2.40E-03	-1.68	44	0.55	LOC122441558	
6:11684001	6	11684001	11685000	1000	1	3.42E-05	1.72E-02	-1.059	3	0.3	DENND4A	
6:32641001	6	32641001	32642000	1000	1	2.97E-05	1.70E-02	-1.062	2	0.2		
6:36391001	6	36391001	36392000	1000	1	8.57E-05	2.55E-02	-1.219	2	0.2	LOC122444281	
6:88581001	6	88581001	88583000	2000	1	8.28E-05	2.51E-02	1.113	90	4.5		
7:60046001	7	60046001	60048000	2000	1	7.30E-05	2.36E-02	-0.955	13	0.65		
7:68141001	7	68141001	68142000	1000	1	1.63E-05	1.29E-02	-1.302	6	0.6	NLGN1	Cytoskeleton
8:28811001	8	28811001	28812000	1000	1	6.88E-06	8.96E-03	-1.209	7	0.7	KAZALD1	
8:41717001	8	41717001	41718000	1000	1	3.31E-05	1.72E-02	-1.3	3	0.3	LOC122446204;LOC122446962	
8:80103001	8	80103001	80106000	3000	1	6.98E-05	2.35E-02	-1.234	32	1.07	RET	Receptor
8:89177001	8	89177001	89179000	2000	1	1.22E-05	1.11E-02	-1.275	8	0.4		
8:90232001	8	90232001	90233000	1000	1	3.60E-05	1.74E-02	-0.941	3	0.3		
9:63404001	9	63404001	63406000	2000	1	2.74E-05	1.65E-02	-1.353	13	0.65		
9:69485001	9	69485001	69486000	1000	1	1.12E-05	1.09E-02	-0.966	16	1.6	CCDC70	
10:30601001	10	30601001	30602000	1000	1	6.08E-06	8.44E-03	-1.063	5	0.5	FRMD4A	
10:69299001	10	69299001	69301000	2000	1	6.00E-05	2.17E-02	-1.035	10	0.5		
11:221001	11	221001	223000	2000	1	4.44E-05	1.91E-02	-1.256	6	0.3		
11:8545001	11	8545001	8547000	2000	1	8.41E-05	2.53E-02	-1.151	10	0.5	CNTN5	
11:72959001	11	72959001	72960000	1000	1	4.56E-05	1.93E-02	-1.094	5	0.5	LOC122450481;LOC122450573;LOC122450570	
11:74547001	11	74547001	74549000	2000	1	8.30E-08	1.45E-03	-1.25	11	0.55	LOC122450482	
12:8330001	12	8330001	8356000	26000	10	1.99E-06	5.71E-03	-2.033	199	0.77	LOC122451337	
12:8359001	12	8359001	8376000	17000	7	5.80E-08	1.25E-03	-2.199	148	0.87	LOC122451337	
12:8377001	12	8377001	8430000	53000	27	3.85E-09	3.46E-04	-2.256	339	0.64		
12:15412001	12	15412001	15415000	3000	2	2.33E-05	1.53E-02	-1.658	37	1.23	LOC122451178;LOC122451183	
12:15472001	12	15472001	15473000	1000	1	3.35E-05	1.72E-02	-1.095	7	0.7	LOC122451183;LOC122451564	
12:52336001	12	52336001	52337000	1000	1	1.59E-05	1.29E-02	-1.153	3	0.3		
12:68792001	12	68792001	68793000	1000	1	1.16E-06	4.82E-03	-1.102	4	0.4		
12:75715001	12	75715001	75718000	3000	1	7.63E-05	2.42E-02	-1.043	28	0.93		
14:22364001	14	22364001	22365000	1000	1	3.73E-06	7.46E-03	-1.074	3	0.3		
14:41249001	14	41249001	41250000	1000	1	1.04E-07	1.48E-03	-1.653	5	0.5	FMO2	Metabolism
14:62996001	14	62996001	63001000	5000	2	6.50E-05	2.26E-02	-1.035	42	0.84	LOC122453050	
14:63839001	14	63839001	63841000	2000	1	9.99E-05	2.75E-02	-1.33	26	1.3		
14:63889001	14	63889001	63891000	2000	1	5.78E-05	2.14E-02	-1.382	27	1.35	LOC122453376;LOC122453375	
14:63910001	14	63910001	63911000	1000	1	3.02E-06	6.71E-03	-1.428	12	1.2	LOC122453375	
14:63925001	14	63925001	63928000	3000	1	8.18E-05	2.51E-02	-1.618	37	1.23		
14:64052001	14	64052001	64058000	6000	2	5.16E-07	3.37E-03	-1.75	44	0.73		
14:67420001	14	67420001	67423000	3000	2	2.82E-05	1.66E-02	-1.271	28	0.93		
14:67489001	14	67489001	67493000	4000	2	4.16E-05	1.87E-02	-1.41	20	0.5		
14:67660001	14	67660001	67661000	1000	1	2.74E-05	1.65E-02	-1.684	11	1.1		
14:67733001	14	67733001	67736000	3000	1	2.56E-08	6.68E-04	-1.378	14	0.47	LOC122453329	
14:67743001	14	67743001	67745000	2000	1	1.80E-06	5.54E-03	-1.917	18	0.9	LOC122453329	
14:67749001	14	67749001	67772000	23000	12	1.33E-07	1.74E-03	-2.067	224	0.97	LOC122453329	
14:67774001	14	67774001	67781000	7000	1	3.08E-06	6.71E-03	-1.564	70	1		
14:67897001	14	67897001	67899000	2000	1	2.41E-05	1.54E-02	-1.377	84	4.2		
14:67946001	14	67946001	67949000	3000	3	1.55E-06	5.20E-03	-1.805	8	0.27		

14:67966001	14	67966001	67970000	4000	4	4.89E-06	7.59E-03	-1.778	17	0.42		
14:67994001	14	67994001	67999000	5000	2	1.17E-06	4.82E-03	-1.625	43	0.86	LOC122453182	
14:68001001	14	68001001	68008000	7000	4	6.36E-08	1.25E-03	-2.058	22	0.31	LOC122453182	
14:68020001	14	68020001	68021000	1000	1	1.21E-05	1.11E-02	-1.268	5	0.5		
14:68054001	14	68054001	68057000	3000	2	4.57E-06	7.46E-03	-1.312	19	0.63		
14:70526001	14	70526001	70528000	2000	1	1.91E-05	1.41E-02	-1.745	33	1.65		
14:70540001	14	70540001	70546000	6000	1	8.37E-05	2.52E-02	-1.291	61	1.02		
14:70702001	14	70702001	70704000	2000	1	4.11E-06	7.46E-03	-1.987	12	0.6	LOC122453089;TRNAE-UUC	
14:70706001	14	70706001	70720000	14000	1	1.17E-05	1.09E-02	-1.69	139	0.99	LOC122453089;TRNAE-UUC	
14:70746001	14	70746001	70747000	1000	1	2.13E-05	1.46E-02	-1.48	14	1.4	LOC122453088;LOC122453093;TRNAE-UUC	
14:70762001	14	70762001	70769000	7000	1	7.49E-05	2.40E-02	-1.456	81	1.16	LOC122453093;TRNAE-UUC;LOC122453092	
14:70776001	14	70776001	70784000	8000	1	5.10E-05	2.02E-02	-1.434	86	1.07	LOC122453093;LOC122453092	
14:70836001	14	70836001	70840000	4000	1	3.55E-05	1.73E-02	-1.562	52	1.3	LOC122453091	
14:70920001	14	70920001	70931000	11000	1	6.78E-06	8.94E-03	-1.57	81	0.74	LOC122453090	
14:70932001	14	70932001	70935000	3000	1	2.12E-05	1.46E-02	-1.756	25	0.83		
14:70937001	14	70937001	70941000	4000	1	3.17E-05	1.72E-02	-1.429	31	0.78	LOC122453072	
14:71054001	14	71054001	71057000	3000	2	4.04E-06	7.46E-03	-1.653	24	0.8	TRNAE-UUC;LOC122452908	
14:71078001	14	71078001	71085000	7000	2	9.38E-05	2.66E-02	-1.302	37	0.53		
14:72427001	14	72427001	72429000	2000	1	8.56E-07	4.65E-03	1.841	158	7.9		
14:73040001	14	73040001	73041000	1000	1	1.12E-05	1.09E-02	-1.577	11	1.1	TRNAC-GCA	
14:73783001	14	73783001	73790000	7000	1	5.41E-05	2.06E-02	-1.439	305	4.36		
14:73876001	14	73876001	73882000	6000	2	2.62E-05	1.61E-02	-1.456	278	4.63		
14:73883001	14	73883001	73888000	5000	1	4.89E-05	1.97E-02	-1.461	215	4.3		
14:73893001	14	73893001	73905000	12000	4	8.60E-07	4.65E-03	-1.689	549	4.58		
15:51621001	15	51621001	51626000	5000	2	6.31E-05	2.24E-02	-1.597	57	1.14		
15:51627001	15	51627001	51635000	8000	2	3.58E-05	1.73E-02	-1.716	81	1.01		
15:71541001	15	71541001	71543000	2000	1	6.43E-05	2.26E-02	-0.953	10	0.5		
16:33447001	16	33447001	33448000	1000	1	3.44E-05	1.72E-02	-1.17	2	0.2	C9;LOC122454673	
17:4573001	17	4573001	4574000	1000	1	3.24E-06	6.95E-03	1.399	29	2.9		
18:7304001	18	7304001	7305000	1000	1	8.88E-05	2.60E-02	-1.029	1	0.1		
18:9392001	18	9392001	9395000	3000	1	1.31E-05	1.17E-02	-1.06	30	1	LOC122420697;LOC122420701	
18:9735001	18	9735001	9737000	2000	1	7.07E-05	2.36E-02	-1.47	14	0.7	LOC122420399;LOC122420592;LOC122420668	
18:10200001	18	10200001	10201000	1000	1	1.82E-05	1.38E-02	-1.563	9	0.9	LOC122420582	
18:19495001	18	19495001	19496000	1000	1	9.46E-05	2.68E-02	-0.814	58	5.8	BICRA;EHD2	Transport
18:41743001	18	41743001	41744000	1000	1	6.05E-05	2.19E-02	1.113	28	2.8	LOC122420767;SLC6A2	Transport
19:32105001	19	32105001	32106000	1000	1	7.29E-05	2.36E-02	-1.002	2	0.2	GRID2	Receptor
19:60183001	19	60183001	60184000	1000	1	3.71E-05	1.76E-02	-1.189	2	0.2	QRFPR;TRNAC-ACA	Signaling
20:9603001	20	9603001	9604000	1000	1	3.00E-05	1.70E-02	-1.1	6	0.6		
20:18489001	20	18489001	18490000	1000	1	2.85E-05	1.67E-02	-1.105	0	0	LOC122422953	
20:22668001	20	22668001	22670000	2000	1	3.27E-05	1.72E-02	-1.239	12	0.6		
20:22715001	20	22715001	22718000	3000	1	3.04E-05	1.72E-02	-1.562	8	0.27	LOC122422605	
20:22719001	20	22719001	22721000	2000	1	9.18E-05	2.63E-02	-1.124	13	0.65	LOC122422605	
20:22778001	20	22778001	22789000	11000	1	4.72E-05	1.96E-02	-1.491	104	0.95	LOC122422603;LOC122422612;LOC122422611;LOC122422697	
20:22827001	20	22827001	22829000	2000	1	6.75E-05	2.32E-02	-1.247	15	0.75	LOC122422607	
20:22849001	20	22849001	22856000	7000	3	3.32E-05	1.72E-02	-1.41	43	0.61	LOC122422698;LOC122422610	
20:29824001	20	29824001	29825000	1000	1	6.96E-07	4.14E-03	-1.158	1	0.1		
20:30884001	20	30884001	30885000	1000	1	8.84E-05	2.60E-02	-1.342	1	0.1		
20:43634001	20	43634001	43635000	1000	1	6.34E-05	2.24E-02	-0.94	9	0.9		
21:9656001	21	9656001	9657000	1000	1	1.62E-05	1.29E-02	-1.061	16	1.6	PLA2G6	Metabolism
22:10873001	22	10873001	10875000	2000	1	5.97E-05	2.17E-02	-0.912	23	1.15	MAPKAPK3	Signaling
22:23616001	22	23616001	23618000	2000	1	5.45E-05	2.07E-02	-1.644	12	0.6	LOC122424520	
22:23620001	22	23620001	23626000	6000	1	2.39E-06	6.03E-03	-1.614	63	1.05	LOC122424520	
23:2019001	23	2019001	2020000	1000	1	5.79E-05	2.14E-02	1.103	14	1.4	RNF165	Proteolysis
23:53236001	23	53236001	53238000	2000	1	6.13E-05	2.19E-02	-1.246	12	0.6	LOC122425820	
24:5904001	24	5904001	5905000	1000	1	7.81E-05	2.43E-02	1.076	28	2.8	EPHB2	Receptor
24:30153001	24	30153001	30154000	1000	1	8.13E-05	2.51E-02	-0.852	4	0.4	LOC122426119	
24:49147001	24	49147001	49148000	1000	1	7.29E-05	2.36E-02	0.982	34	3.4	DNAH7	Cytoskeleton
25:2502001	25	2502001	2515000	13000	2	1.77E-05	1.37E-02	-1.762	133	1.02	LOC122427813	
25:2609001	25	2609001	2610000	1000	1	1.83E-05	1.38E-02	1.106	14	1.4		
25:30458001	25	30458001	30459000	1000	1	2.39E-05	1.54E-02	-0.933	9	0.9	LOC122427759	
25:52942001	25	52942001	52944000	2000	1	3.55E-05	1.73E-02	1.11	19	0.95	TSPAN8	
26:9916001	26	9916001	9936000	20000	2	5.24E-05	2.05E-02	-1.452	734	3.67		
26:10035001	26	10035001	10042000	7000	3	1.15E-05	1.09E-02	-1.583	320	4.57		

26:10075001	26	10075001	10119000	44000	5	5.12E-06	7.71E-03	-1.655	1755	3.99		
26:10120001	26	10120001	10147000	27000	7	1.28E-06	5.13E-03	-1.852	1234	4.57		
26:10148001	26	10148001	10165000	17000	5	4.50E-05	1.92E-02	-1.634	756	4.45		
26:10166001	26	10166001	10268000	102000	23	1.03E-06	4.82E-03	-1.773	4586	4.5		
26:10270001	26	10270001	10281000	11000	4	1.32E-05	1.17E-02	-1.693	484	4.4		
26:10282001	26	10282001	10340000	58000	17	2.17E-06	5.85E-03	-1.794	2539	4.38		
26:10342001	26	10342001	10389000	47000	9	1.03E-06	4.82E-03	-1.831	2088	4.44		
26:10390001	26	10390001	10420000	30000	14	6.99E-06	8.98E-03	-1.713	1369	4.56		
26:10421001	26	10421001	10438000	17000	2	5.17E-05	2.03E-02	-1.447	767	4.51		
26:10441001	26	10441001	10474000	33000	3	4.07E-06	7.46E-03	-1.746	1392	4.22		
26:10690001	26	10690001	10693000	3000	1	6.07E-06	8.44E-03	-1.388	127	4.23		
26:10697001	26	10697001	10701000	4000	1	3.31E-05	1.72E-02	-1.129	156	3.9		
26:10746001	26	10746001	10751000	5000	1	7.90E-05	2.45E-02	-1.187	83	1.66		
26:10774001	26	10774001	10775000	1000	1	1.95E-05	1.42E-02	-0.989	42	4.2		
26:10968001	26	10968001	10971000	3000	1	9.11E-07	4.76E-03	-1.269	122	4.07		
26:11172001	26	11172001	11185000	13000	1	1.59E-06	5.20E-03	-1.789	538	4.14		
26:11447001	26	11447001	11468000	21000	2	3.39E-05	1.72E-02	-1.461	938	4.47		
26:11531001	26	11531001	11533000	2000	1	7.75E-05	2.43E-02	-1.317	89	4.45		
26:11562001	26	11562001	11568000	6000	1	4.44E-06	7.46E-03	-1.556	277	4.62		
26:11570001	26	11570001	11574000	4000	3	4.29E-05	1.88E-02	-1.394	168	4.2		
26:11617001	26	11617001	11624000	7000	3	2.02E-05	1.45E-02	-1.925	318	4.54		
26:12105001	26	12105001	12107000	2000	1	1.41E-05	1.22E-02	-1.498	91	4.55		
26:13513001	26	13513001	13514000	1000	1	1.10E-05	1.09E-02	-1.699	46	4.6		
26:13544001	26	13544001	13547000	3000	1	2.92E-05	1.69E-02	-1.602	122	4.07		
26:13549001	26	13549001	13550000	1000	1	2.04E-05	1.45E-02	-1.558	37	3.7		
26:13680001	26	13680001	13693000	13000	5	3.24E-05	1.72E-02	-1.686	598	4.6		
26:13752001	26	13752001	13753000	1000	1	9.58E-05	2.68E-02	-1.181	9	0.9		
26:13757001	26	13757001	13760000	3000	1	6.75E-05	2.32E-02	-1.285	25	0.83		
26:13761001	26	13761001	13762000	1000	1	4.23E-05	1.87E-02	-1.329	5	0.5		
26:13769001	26	13769001	13770000	1000	1	3.58E-05	1.73E-02	-1.597	5	0.5		
26:13840001	26	13840001	13856000	16000	5	5.99E-06	8.44E-03	-1.697	716	4.47		
26:13861001	26	13861001	13874000	13000	1	1.58E-05	1.29E-02	-1.598	565	4.35		
26:13876001	26	13876001	13890000	14000	2	4.47E-05	1.92E-02	-1.493	620	4.43		
26:13894001	26	13894001	13896000	2000	1	5.38E-05	2.06E-02	-1.489	78	3.9		
26:13897001	26	13897001	13921000	24000	3	4.20E-06	7.46E-03	-1.637	973	4.05		
26:13922001	26	13922001	13925000	3000	2	3.32E-05	1.72E-02	-1.655	144	4.8		
26:13936001	26	13936001	13960000	24000	10	5.98E-06	8.44E-03	-1.756	1112	4.63		
26:13961001	26	13961001	13996000	35000	3	2.39E-06	6.03E-03	-1.699	1562	4.46		
26:13997001	26	13997001	14021000	24000	6	4.55E-06	7.46E-03	-1.684	1053	4.39		
26:14022001	26	14022001	14069000	47000	12	2.10E-06	5.79E-03	-1.799	2123	4.52		
26:14071001	26	14071001	14094000	23000	9	8.18E-06	9.52E-03	-1.758	1052	4.57		
26:14098001	26	14098001	14157000	59000	13	8.65E-06	9.75E-03	-1.715	2667	4.52		
26:14158001	26	14158001	14185000	27000	3	2.63E-05	1.61E-02	-1.61	1123	4.16		
26:14190001	26	14190001	14320000	130000	15	2.37E-07	2.07E-03	-1.757	5421	4.17		
26:14375001	26	14375001	14377000	2000	1	7.16E-05	2.36E-02	-1.482	89	4.45		
26:14387001	26	14387001	14410000	23000	9	2.42E-06	6.03E-03	-1.587	910	3.96		
26:14411001	26	14411001	14426000	15000	2	4.83E-05	1.97E-02	-1.397	603	4.02		
26:14447001	26	14447001	14475000	28000	4	3.25E-05	1.72E-02	-1.673	1102	3.94		
26:14477001	26	14477001	14498000	21000	1	6.64E-06	8.94E-03	-1.523	829	3.95		
26:14514001	26	14514001	14520000	6000	1	2.02E-05	1.45E-02	-1.736	256	4.27		
26:14524001	26	14524001	14529000	5000	1	2.19E-05	1.47E-02	-1.728	214	4.28		
26:14593001	26	14593001	14611000	18000	2	2.58E-05	1.61E-02	-1.806	749	4.16		
26:35984001	26	35984001	35985000	1000	1	7.14E-05	2.36E-02	-1.014	6	0.6	WDFY3	
28:31268001	28	31268001	31270000	2000	1	3.64E-05	1.75E-02	-1.212	7	0.35	LOC122429269	
28:31388001	28	31388001	31391000	3000	1	9.38E-05	2.66E-02	-1.045	29	0.97	LOC122429464	
28:31556001	28	31556001	31558000	2000	1	7.18E-05	2.36E-02	-1.185	16	0.8	LOC122429270	
28:31675001	28	31675001	31677000	2000	1	1.16E-05	1.09E-02	-1.038	23	1.15	LOC122429462;LOC122429474;LOC122429479	
28:31716001	28	31716001	31717000	1000	1	2.10E-05	1.46E-02	-1.227	11	1.1	LOC122429462;LOC122429471	
28:31719001	28	31719001	31720000	1000	1	2.46E-08	6.68E-04	-1.404	2	0.2	LOC122429462;LOC122429471	
28:31902001	28	31902001	31904000	2000	1	3.53E-05	1.73E-02	-1.505	20	1	LOC122429463;LOC122429273	
29:28873001	29	28873001	28875000	2000	1	1.68E-05	1.32E-02	-1.083	16	0.8		
29:35194001	29	35194001	35196000	2000	1	9.84E-06	1.04E-02	-1.134	10	0.5	LOC122430976	
30:22240001	30	22240001	22242000	2000	1	1.07E-05	1.08E-02	1.169	34	1.7		
31:196001	31	196001	199000	3000	1	2.76E-05	1.65E-02	-1.598	18	0.6	LOC122432564;LOC122432262	
31:33008001	31	33008001	33010000	2000	1	7.09E-05	2.36E-02	-0.897	25	1.25		
31:40160001	31	40160001	40163000	3000	1	9.56E-05	2.68E-02	-1.272	28	0.93	LOC122432591	
31:40192001	31	40192001	40195000	3000	1	3.70E-05	1.76E-02	-1.366	49	1.63	LOC122432591	
32:207001	32	207001	209000	2000	1	3.10E-05	1.72E-02	-1.561	21	1.05		

32:226001	32	226001	230000	4000	1	3.09E-05	1.72E-02	-1.561	48	1.2		
32:232001	32	232001	240000	8000	1	1.55E-06	5.20E-03	-1.926	97	1.21	LOC122432858	
32:242001	32	242001	249000	7000	1	2.17E-05	1.47E-02	-1.543	90	1.29	LOC122432858	
32:250001	32	250001	257000	7000	4	1.14E-06	4.82E-03	-2.021	68	0.97	LOC122432858	
32:261001	32	261001	265000	4000	1	1.11E-05	1.09E-02	-1.843	43	1.07	LOC122432858	
32:294001	32	294001	298000	4000	1	1.95E-05	1.42E-02	-1.651	31	0.78	LOC122432858;LOC122432870	
32:305001	32	305001	320000	15000	7	5.10E-06	7.71E-03	-1.747	144	0.96	LOC122432858;LOC122432870	
32:321001	32	321001	337000	16000	3	5.48E-06	8.18E-03	-1.687	185	1.16	LOC122432858;LOC122432870	
32:347001	32	347001	357000	10000	4	4.62E-06	7.46E-03	-1.671	87	0.87	LOC122432858;LOC122432870	
32:359001	32	359001	364000	5000	2	2.59E-07	2.14E-03	-2.157	33	0.66	LOC122432858;LOC122432870	
32:365001	32	365001	395000	30000	12	2.86E-07	2.24E-03	-2.018	203	0.68	LOC122432858;LOC122432870	
32:396001	32	396001	403000	7000	2	1.97E-06	5.71E-03	-1.909	56	0.8	LOC122432858;LOC122432870;LOC122432871	
32:404001	32	404001	425000	21000	6	2.33E-07	2.07E-03	-1.896	239	1.14	LOC122432858;LOC122432870;LOC122432871	
32:427001	32	427001	434000	7000	1	2.49E-06	6.06E-03	-1.081	74	1.06	LOC122432858;LOC122432870	
32:503001	32	503001	522000	19000	7	1.06E-08	5.53E-04	-1.942	165	0.87	LOC122432858;LOC122432867	
32:532001	32	532001	549000	17000	7	1.69E-07	1.77E-03	-2.161	102	0.6	LOC122432858;LOC122432867	
32:550001	32	550001	551000	1000	1	1.01E-07	1.48E-03	-2.205	2	0.2	LOC122432858	
32:552001	32	552001	573000	21000	9	3.98E-07	2.71E-03	-2.141	170	0.81	LOC122432858	
32:575001	32	575001	576000	1000	1	3.21E-05	1.72E-02	-1.591	10	1	LOC122432858	
32:578001	32	578001	582000	4000	1	6.46E-05	2.26E-02	-1.657	29	0.72	LOC122432858	
32:583001	32	583001	585000	2000	1	7.36E-05	2.37E-02	-1.708	18	0.9	LOC122432858	
32:586001	32	586001	588000	2000	1	1.70E-07	1.77E-03	-1.985	11	0.55	LOC122432858	
32:590001	32	590001	595000	5000	2	6.82E-05	2.33E-02	-1.59	39	0.78	LOC122432858	
32:599001	32	599001	602000	3000	1	6.35E-06	8.73E-03	-1.393	34	1.13	LOC122432858	
32:637001	32	637001	647000	10000	3	1.06E-05	1.07E-02	-1.814	429	4.29	LOC122432858	
32:962001	32	962001	965000	3000	1	3.46E-05	1.72E-02	-1.35	27	0.9	LOC122432858;PARN	Translation
32:972001	32	972001	982000	10000	1	3.32E-05	1.72E-02	-1.156	99	0.99	LOC122432858;PARN;LOC122433513	Translation
32:30516001	32	30516001	30518000	2000	1	7.41E-05	2.38E-02	1.313	86	4.3	CPSF4;PTCD1;BUD31;PDAP1	Translation;Metabolism
32:39042001	32	39042001	39043000	1000	1	1.08E-06	4.82E-03	-1.48	5	0.5	LOC122433122	
32:39155001	32	39155001	39160000	5000	1	4.20E-05	1.87E-02	-1.443	46	0.92	LOC122433075	
32:39193001	32	39193001	39200000	7000	1	7.75E-05	2.43E-02	-1.297	43	0.61	LOC122433150	
X:492001	X	492001	494000	2000	1	5.04E-05	2.01E-02	-1.103	21	1.05	LOC122434384	
X:582001	X	582001	584000	2000	1	8.16E-06	9.52E-03	-1.299	15	0.75		
X:690001	X	690001	691000	1000	1	8.73E-05	2.58E-02	-1.597	29	2.9		
X:3926001	X	3926001	3928000	2000	2	1.39E-06	5.20E-03	-1.622	18	0.9	LOC122434705	
X:19197001	X	19197001	19199000	2000	1	1.42E-06	5.20E-03	-1.229	12	0.6	GRPR	Receptor
X:38419001	X	38419001	38420000	1000	1	6.09E-05	2.19E-02	-0.916	10	1	LOC122434641;LOC122435520	
X:50212001	X	50212001	50213000	1000	1	7.49E-06	9.38E-03	-1.558	11	1.1		
X:62059001	X	62059001	62060000	1000	1	2.99E-05	1.70E-02	-1.017	9	0.9		
X:75550001	X	75550001	75551000	1000	1	2.60E-05	1.61E-02	-1.077	2	0.2	RPS6KA6	Golgi
X:78825001	X	78825001	78827000	2000	1	7.86E-06	9.48E-03	-0.881	12	0.6		
X:86907001	X	86907001	86908000	1000	1	8.98E-05	2.61E-02	-1.33	3	0.3		
X:88005001	X	88005001	88006000	1000	1	1.46E-05	1.24E-02	-1.282	0	0		
X:97589001	X	97589001	97602000	13000	1	3.46E-05	1.72E-02	-1.198	88	0.68	LOC122435661;LOC122435662	
X:99362001	X	99362001	99363000	1000	1	7.06E-06	9.00E-03	-1.56	6	0.6	LOC122436024	
X:99486001	X	99486001	99487000	1000	1	1.38E-05	1.21E-02	-1.611	3	0.3	LOC122435671	
X:99612001	X	99612001	99615000	3000	1	4.42E-09	3.46E-04	-1.584	28	0.93	LOC122435824	
X:99617001	X	99617001	99620000	3000	2	5.13E-05	2.03E-02	-1.284	20	0.67	LOC122435824;LOC122435672;LOC122435863	
X:99647001	X	99647001	99652000	5000	2	7.12E-07	4.14E-03	-1.475	40	0.8	LOC122435863	
X:101275001	X	101275001	101276000	1000	1	6.76E-06	8.94E-03	-1.19	5	0.5		
X:103092001	X	103092001	103093000	1000	1	8.82E-06	9.75E-03	-1.085	1	0.1		
X:105718001	X	105718001	105719000	1000	1	1.47E-05	1.24E-02	-1.415	5	0.5	LOC122434292	
X:120006001	X	120006001	120007000	1000	1	6.07E-05	2.19E-02	-1.073	6	0.6		
X:139087001	X	139087001	139090000	3000	1	3.42E-05	1.72E-02	-1.259	31	1.03		
X:139151001	X	139151001	139156000	5000	1	1.63E-05	1.29E-02	-1.265	54	1.08	LOC122435756	
X:139157001	X	139157001	139161000	4000	1	8.32E-06	9.59E-03	-1.393	46	1.15	LOC122435756	
X:145632001	X	145632001	145633000	1000	1	2.58E-05	1.61E-02	-1.776	34	3.4		
Y:361001	Y	361001	369000	8000	4	8.91E-06	9.76E-03	-1.638	51	0.64		
Y:377001	Y	377001	381000	4000	1	2.95E-05	1.70E-02	-1.33	22	0.55		

Supplemental Table S2. Rocky Mountain Female Elk DMR p<1e-04. DMR name, chromosome number, start nucleotide site, length (bp), p-value, maximum log fold change (LFC), CpG number and density, gene annotation, and gene category.

Supplemental Table S3
Roosevelt Female DMR p<1e-04

DMR Name	Chr	Start	Stop	Length	# Sig Win	minP	minFDR	maxLFC	CpG #/ 1Kb	CpG Density/ 100 bp	Gene Annotation	Gene Category
1:5155001	1	5155001	5156000	1000	1	4.85E-05	2.66E-02	0.55	43	4.3	C1H17orf80	
1:11318001	1	11318001	11319000	1000	1	8.52E-05	3.15E-02	0.589	19	1.9	LOC122438278;LOC122438272	
1:13075001	1	13075001	13076000	1000	1	4.14E-07	4.02E-03	0.841	28	2.8	METRNL	
1:32979001	1	32979001	32980000	1000	1	9.39E-06	1.45E-02	0.602	22	2.2	RNF227;CHD3;LOC122425693	
1:34060001	1	34060001	34061000	1000	1	4.34E-05	2.59E-02	0.576	29	2.9	SPAG7;CAMTA2;INCA1	Metabolism;Transcription
1:35044001	1	35044001	35046000	2000	1	5.00E-05	2.66E-02	0.739	37	1.85	PITPNM3;TRNAK-UUU	Metabolism
1:37264001	1	37264001	37268000	4000	1	4.74E-05	2.66E-02	0.58	75	1.88	SMG6	Metabolism
1:40855001	1	40855001	40856000	1000	1	5.25E-06	1.06E-02	0.797	16	1.6	NOS2	Metabolism
1:46760001	1	46760001	46762000	2000	1	2.09E-06	8.13E-03	0.737	83	4.15	ACACA	
1:56274001	1	56274001	56277000	3000	1	9.41E-05	3.21E-02	-1.062	13	0.43		
1:57031001	1	57031001	57033000	2000	1	2.65E-05	2.18E-02	-1.146	9	0.45		
1:67822001	1	67822001	67823000	1000	1	2.97E-05	2.31E-02	-0.935	4	0.4		
1:90932001	1	90932001	90933000	1000	1	2.11E-05	2.03E-02	-0.691	5	0.5	LOC122424829	
1:97968001	1	97968001	97970000	2000	1	1.18E-05	1.56E-02	-0.878	6	0.3		
1:98394001	1	98394001	98395000	1000	1	2.48E-05	2.16E-02	-0.95	4	0.4		
1:107308001	1	107308001	107309000	1000	1	9.40E-06	1.45E-02	0.72	51	5.1	TANGO2	
1:107436001	1	107436001	107437000	1000	1	8.71E-05	3.15E-02	0.776	53	5.3	GNB1L;TBX1	Cytoskeleton;Transcription
1:107668001	1	107668001	107670000	2000	1	4.22E-05	2.56E-02	0.72	101	5.05	GSC2;ESS2;TSSK2;TSSK1B	Development;Signaling
1:107699001	1	107699001	107701000	2000	1	1.66E-06	7.89E-03	1.099	25	1.25	DGCR2	
1:107835001	1	107835001	107837000	2000	2	2.32E-05	2.10E-02	1.061	52	2.6	MED15;LOC122455045	
1:107841001	1	107841001	107845000	4000	1	6.36E-05	2.86E-02	0.754	243	6.08	MED15;LOC122455045	
1:108187001	1	108187001	108190000	3000	1	6.52E-05	2.89E-02	0.795	155	5.17	LOC122455267;PPIL2;YPEL1	Transcription
1:109731001	1	109731001	109732000	1000	1	5.13E-05	2.67E-02	-1.047	4	0.4	LOC122440814	
1:111835001	1	111835001	111836000	1000	1	6.12E-07	4.58E-03	0.791	33	3.3	LOC122421479;KREMEN1	Receptor
1:117452001	1	117452001	117457000	5000	1	5.19E-05	2.67E-02	0.771	293	5.86	PXN;RPLP0;GCN1	Cytoskeleton;Translation;Cytoskeleton
1:125017001	1	125017001	125018000	1000	1	1.97E-05	1.98E-02	0.656	34	3.4	LOC122423984	
1:126797001	1	126797001	126800000	3000	1	9.52E-05	3.21E-02	0.665	56	1.87	NCOR2	Epigenetic
2:4286001	2	4286001	4287000	1000	1	6.86E-06	1.18E-02	-0.72	8	0.8		
2:15598001	2	15598001	15601000	3000	1	4.99E-05	2.66E-02	0.745	91	3.03	CRTC2;DENND4B;LOC122433353	Transcription
2:21662001	2	21662001	21663000	1000	1	3.23E-05	2.38E-02	-0.792	8	0.8		
2:29492001	2	29492001	29494000	2000	1	7.23E-05	2.98E-02	0.759	53	2.65	RHOC;MOV10	Signaling
2:35224001	2	35224001	35225000	1000	1	3.40E-05	2.39E-02	-1.124	2	0.2	LOC122436613	
2:36926001	2	36926001	36928000	2000	1	8.09E-05	3.13E-02	-0.783	20	1		
2:40362001	2	40362001	40363000	1000	1	5.46E-05	2.71E-02	-0.743	7	0.7	LOC122429391	
2:42831001	2	42831001	42832000	1000	1	4.15E-05	2.55E-02	-1.161	3	0.3		
2:58006001	2	58006001	58007000	1000	1	8.61E-05	3.15E-02	-0.687	9	0.9		
2:58981001	2	58981001	58983000	2000	1	9.37E-05	3.21E-02	-0.793	11	0.55		
2:65484001	2	65484001	65486000	2000	1	1.89E-05	1.93E-02	-0.648	14	0.7	SLC44A5	Transport
2:70333001	2	70333001	70334000	1000	1	1.56E-05	1.81E-02	-0.843	7	0.7	PTGER3	Signaling
2:88783001	2	88783001	88784000	1000	1	2.64E-05	2.18E-02	-0.87	4	0.4	ZYG11B;LOC122437418	
2:94079001	2	94079001	94080000	1000	1	5.15E-05	2.67E-02	-0.961	4	0.4	TRNAE-CUC;LOC122437017	
2:95449001	2	95449001	95450000	1000	1	9.94E-05	3.28E-02	-0.765	5	0.5	MMACHC;CCDC163;TESK2;LOC122437469	
2:114175001	2	114175001	114176000	1000	1	3.99E-06	9.68E-03	1.125	30	3	CROCC2	Epigenetic
3:9370001	3	9370001	9371000	1000	1	1.18E-05	1.56E-02	-0.731	4	0.4		
3:12155001	3	12155001	12156000	1000	1	1.26E-05	1.63E-02	-0.764	5	0.5	DYNC1I1	Cytoskeleton
3:16761001	3	16761001	16762000	1000	1	6.76E-05	2.91E-02	-0.762	6	0.6		
3:17250001	3	17250001	17253000	3000	1	6.62E-05	2.90E-02	-0.894	15	0.5		
3:24587001	3	24587001	24588000	1000	1	2.61E-05	2.18E-02	-0.8	10	1	SNX13	Cytoskeleton
3:31673001	3	31673001	31674000	1000	1	6.47E-05	2.87E-02	-0.75	8	0.8	GRM3	Signaling
3:39454001	3	39454001	39455000	1000	1	3.86E-05	2.48E-02	-0.826	3	0.3	MAGI2	
3:48083001	3	48083001	48084000	1000	1	1.47E-05	1.77E-02	-1.095	3	0.3	ASZ1	
3:51894001	3	51894001	51896000	2000	1	3.46E-06	9.28E-03	-0.955	5	0.25	LOC122438359	
3:53494001	3	53494001	53495000	1000	1	9.71E-05	3.24E-02	-0.826	3	0.3		
3:61799001	3	61799001	61801000	2000	1	1.02E-05	1.48E-02	0.723	46	2.3	MINDY4;LOC122438905	
3:62159001	3	62159001	62161000	2000	1	5.03E-05	2.66E-02	-0.955	16	0.8	NOD1	Cytoskeleton
3:65119001	3	65119001	65121000	2000	1	7.34E-05	2.99E-02	0.69	89	4.45	HOXA9;HOXA7;HOXA6;HOXA5	Development
3:89494001	3	89494001	89495000	1000	1	1.31E-05	1.67E-02	-0.762	9	0.9	TSGA13	
3:92416001	3	92416001	92419000	3000	1	3.00E-05	2.31E-02	-1.242	15	0.5	EXOC4	Transport
3:100375001	3	100375001	100376000	1000	1	9.50E-06	1.45E-02	-0.866	8	0.8	LOC122438738	
3:106155001	3	106155001	106156000	1000	1	6.29E-05	2.86E-02	-0.703	6	0.6	CNTNAP2	
4:18007001	4	18007001	18009000	2000	1	2.04E-05	2.01E-02	-1.118	13	0.65		
4:24930001	4	24930001	24931000	1000	1	9.12E-05	3.19E-02	-0.834	5	0.5		
4:27521001	4	27521001	27522000	1000	1	1.87E-05	1.92E-02	-0.847	13	1.3		
4:39109001	4	39109001	39110000	1000	1	3.98E-05	2.51E-02	-0.81	5	0.5		
4:41779001	4	41779001	41780000	1000	1	5.85E-05	2.78E-02	-0.886	6	0.6		
4:53238001	4	53238001	53239000	1000	1	3.33E-05	2.39E-02	-0.73	4	0.4		

4:65890001	4	65890001	65891000	1000	1	7.29E-05	2.98E-02	-1.229	5	0.5		
4:68028001	4	68028001	68029000	1000	1	2.76E-05	2.20E-02	-0.725	44	4.4	MGAT1	Golgi
4:69664001	4	69664001	69665000	1000	1	5.81E-05	2.78E-02	0.725	35	3.5	HK3;UNC5A	Signaling;Receptor
4:73861001	4	73861001	73863000	2000	1	1.31E-05	1.67E-02	-0.836	10	0.5		
4:84662001	4	84662001	84664000	2000	1	5.49E-05	2.71E-02	-0.921	9	0.45	MEIKIN	
4:86883001	4	86883001	86884000	1000	1	5.83E-05	2.78E-02	0.594	29	2.9	CACTIN;PIP5K1C	Cytoskeleton;Signaling
4:90354001	4	90354001	90356000	2000	1	2.97E-07	3.85E-03	0.83	44	2.2	TIMM44;CTXN1;SNAPC2;TGFBR3L;<br>MAP2K7	Transport;Signaling
4:90368001	4	90368001	90372000	4000	1	7.31E-05	2.98E-02	0.828	133	3.33	SNAPC2;TGFBR3L;MAP2K7;LRRC8E;<br>PRR36	Signaling;Cytoskeleton
4:92342001	4	92342001	92344000	2000	1	8.31E-05	3.13E-02	0.756	88	4.4	EIF3G;P2RY11;PPAN;LOC122441153;<br>LOC122441144;ANGPTL6	Translation;Signaling;Metabolism;Signaling
4:104515001	4	104515001	104518000	3000	1	5.54E-05	2.71E-02	0.681	108	3.6	CRTC1;LOC122440769	Transcription
4:104819001	4	104819001	104820000	1000	1	4.63E-05	2.66E-02	0.751	44	4.4	SLC25A42	Transport
4:104971001	4	104971001	104972000	1000	1	5.48E-07	4.26E-03	0.767	24	2.4	SUGP1;MAU2	Translation
5:15690001	5	15690001	15693000	3000	1	1.55E-05	1.81E-02	-1.17	17	0.57		
5:19332001	5	19332001	19333000	1000	1	1.53E-05	1.81E-02	-0.915	5	0.5		
5:45156001	5	45156001	45158000	2000	1	4.91E-05	2.66E-02	-1.079	8	0.4	REEP1	Transport
5:48408001	5	48408001	48410000	2000	1	4.18E-05	2.55E-02	-0.885	14	0.7		
5:48921001	5	48921001	48923000	2000	1	1.26E-06	6.82E-03	-1.139	7	0.35		
5:51166001	5	51166001	51167000	1000	1	1.68E-05	1.83E-02	-0.917	3	0.3		
5:60614001	5	60614001	60615000	1000	1	7.78E-06	1.28E-02	-1.119	3	0.3		
5:72428001	5	72428001	72430000	2000	1	3.71E-05	2.42E-02	-0.816	19	0.95		
5:91016001	5	91016001	91018000	2000	1	3.14E-05	2.35E-02	0.752	17	0.85	ARPC5L;GOLGA1	Cytoskeleton
5:94727001	5	94727001	94729000	2000	1	4.95E-05	2.66E-02	0.672	49	2.45		
5:94771001	5	94771001	94773000	2000	1	6.65E-05	2.90E-02	0.77	46	2.3		
5:98013001	5	98013001	98016000	3000	1	6.05E-06	1.14E-02	0.771	124	4.13	CEL;RALGDS	Cytoskeleton;Transcription
5:98017001	5	98017001	98019000	2000	1	4.57E-05	2.66E-02	0.822	80	4	RALGDS	Transcription
5:98430001	5	98430001	98432000	2000	1	7.00E-07	5.04E-03	1.029	101	5.05	NACC2	
5:98748001	5	98748001	98749000	1000	1	7.27E-05	2.98E-02	0.764	60	6	DNLZ;CARD9;SNAPC4	
5:98780001	5	98780001	98783000	3000	1	8.08E-05	3.13E-02	0.67	100	3.33	SNAPC4;ENTR1;PMPCA;INPP5E	Protease;Signaling
5:98870001	5	98870001	98873000	3000	1	3.37E-05	2.39E-02	0.842	174	5.8	NOTCH1	
5:99525001	5	99525001	99527000	2000	1	3.93E-05	2.49E-02	0.634	84	4.2	VAV2	
5:101042001	5	101042001	101044000	2000	1	9.40E-05	3.21E-02	0.863	104	5.2	ABCA2;CLIC3	Transport
5:101543001	5	101543001	101545000	2000	1	1.60E-05	1.81E-02	0.832	87	4.35	EHMT1	
6:1403001	6	1403001	1404000	1000	1	5.03E-05	2.66E-02	-0.671	6	0.6	EPB41L4A;LOC122443995;LOC12244<br>4454	
6:5050001	6	5050001	5052000	2000	1	2.67E-05	2.18E-02	-0.773	12	0.6		
6:9526001	6	9526001	9527000	1000	1	7.59E-05	3.03E-02	-0.846	11	1.1	HOMER1	
6:10675001	6	10675001	10676000	1000	1	2.48E-05	2.16E-02	-0.848	5	0.5	GPR137C	
6:28757001	6	28757001	28758000	1000	1	2.34E-07	3.25E-03	-0.884	3	0.3		
6:34529001	6	34529001	34530000	1000	1	5.20E-05	2.67E-02	-1.2	3	0.3	MDGA2	Immune
6:40432001	6	40432001	40434000	2000	1	8.48E-05	3.15E-02	0.671	36	1.8	TRIM9	Proteolysis
6:48491001	6	48491001	48493000	2000	1	9.71E-06	1.46E-02	-1.071	5	0.25	ALDH1A2	Metabolism
6:52794001	6	52794001	52795000	1000	1	4.22E-05	2.56E-02	-0.764	6	0.6		
6:62628001	6	62628001	62629000	1000	1	2.99E-05	2.31E-02	0.576	13	1.3	SAMD4A	
6:63612001	6	63612001	63615000	3000	1	8.96E-05	3.17E-02	-1.047	18	0.6		
6:70006001	6	70006001	70007000	1000	1	3.35E-05	2.39E-02	-0.757	4	0.4	KCNH5	Transport
6:75943001	6	75943001	75945000	2000	1	4.21E-05	2.56E-02	0.586	24	1.2	GALNT16	Golgi
7:6494001	7	6494001	6495000	1000	1	2.61E-05	2.18E-02	-0.712	7	0.7		
7:10208001	7	10208001	10209000	1000	1	2.10E-05	2.03E-02	-0.789	7	0.7	STAG1	Epigenetic
7:12796001	7	12796001	12798000	2000	1	7.50E-05	3.03E-02	-0.633	12	0.6		
7:33608001	7	33608001	33609000	1000	1	1.29E-05	1.66E-02	-1.097	1	0.1	EFHB	Signaling
7:37179001	7	37179001	37180000	1000	1	9.49E-05	3.21E-02	-0.751	5	0.5	GAP43	
7:42213001	7	42213001	42215000	2000	1	8.76E-05	3.15E-02	-0.996	9	0.45		
7:43945001	7	43945001	43947000	2000	1	8.68E-05	3.15E-02	0.58	25	1.25	PDIA5	Transcription
7:46015001	7	46015001	46017000	2000	1	4.28E-05	2.58E-02	-0.813	13	0.65	SLC12A8	Transport
7:47040001	7	47040001	47042000	2000	1	6.09E-06	1.14E-02	0.667	60	3	TNK2	
7:52310001	7	52310001	52311000	1000	1	5.93E-05	2.79E-02	-0.858	2	0.2		
7:61546001	7	61546001	61547000	1000	1	2.34E-07	3.25E-03	-1.191	4	0.4		
7:61924001	7	61924001	61927000	3000	1	1.72E-05	1.85E-02	-1.227	20	0.67		
7:63770001	7	63770001	63772000	2000	1	8.75E-05	3.15E-02	-0.716	10	0.5	KCNMB2	
7:66067001	7	66067001	66069000	2000	1	8.76E-05	3.15E-02	-0.962	4	0.2		
7:76293001	7	76293001	76294000	1000	1	7.58E-05	3.03E-02	-0.806	13	1.3	LOC122444969;LOC122444967	
7:78721001	7	78721001	78722000	1000	1	2.89E-06	8.54E-03	-0.712	7	0.7		
8:1000001	8	1000001	1004000	4000	1	2.37E-05	2.11E-02	0.686	106	2.65	NKX6-2;INPP5A	Development;Signaling
8:1279001	8	1279001	1281000	2000	1	4.92E-05	2.66E-02	0.682	124	6.2	LRRC27	
8:3526001	8	3526001	3527000	1000	1	6.00E-05	2.80E-02	-0.767	24	2.4		
8:33829001	8	33829001	33833000	4000	1	2.07E-05	2.02E-02	-0.625	52	1.3	LOC122446562	
8:35490001	8	35490001	35491000	1000	1	1.60E-05	1.81E-02	-0.845	2	0.2	FFAR4	Signaling
8:49921001	8	49921001	49922000	1000	1	2.59E-05	2.18E-02	-0.77	10	1		
8:63225001	8	63225001	63226000	1000	1	1.59E-05	1.81E-02	-0.848	7	0.7	ADK	

8:69475001	8	69475001	69476000	1000	1	9.38E-05	3.21E-02	-1.132	4	0.4	CTNNA3	Cytoskeleton
8:75865001	8	75865001	75866000	1000	1	4.43E-06	9.92E-03	-0.87	2	0.2	TMEM26	
8:78863001	8	78863001	78865000	2000	1	7.02E-05	2.95E-02	-0.742	15	0.75	BICC1;LOC122446553	Metabolism
8:82254001	8	82254001	82255000	1000	1	4.03E-05	2.53E-02	-0.879	2	0.2		
8:90538001	8	90538001	90539000	1000	1	9.55E-05	3.21E-02	0.59	22	2.2	GALNT2	Golgi
8:91533001	8	91533001	91535000	2000	1	1.67E-05	1.82E-02	-0.722	8	0.4		
9:1007001	9	1007001	1009000	2000	1	6.17E-05	2.82E-02	0.723	115	5.75	TUBGCP3	Cytoskeleton
9:1978001	9	1978001	1980000	2000	1	9.28E-05	3.20E-02	0.873	117	5.85	CARS2;NAXD;LOC122447113;LOC122447586	Translation;Metabolism
9:17209001	9	17209001	17210000	1000	1	8.67E-05	3.15E-02	-1.048	4	0.4	LOC122447240;LOC122447589	
9:21420001	9	21420001	21422000	2000	1	5.44E-05	2.71E-02	-1.091	8	0.4	GPC5	
9:24406001	9	24406001	24408000	2000	1	5.00E-05	2.66E-02	-0.703	15	0.75		
9:29955001	9	29955001	29957000	2000	1	3.44E-05	2.39E-02	-0.688	18	0.9		
9:43267001	9	43267001	43268000	1000	1	8.06E-05	3.13E-02	-1.111	4	0.4		
9:51146001	9	51146001	51148000	2000	1	7.65E-05	3.04E-02	-0.804	11	0.55	LOC122447273	
9:53636001	9	53636001	53637000	1000	1	8.48E-05	3.15E-02	-0.745	4	0.4		
9:54326001	9	54326001	54328000	2000	1	5.54E-05	2.71E-02	-0.733	9	0.45		
9:54536001	9	54536001	54538000	2000	1	8.77E-05	3.15E-02	-1.068	12	0.6		
9:63405001	9	63405001	63406000	1000	1	8.63E-07	5.41E-03	-1.077	10	1		
9:67360001	9	67360001	67361000	1000	1	7.08E-05	2.96E-02	0.671	42	4.2	LOC122447612	
9:82895001	9	82895001	82896000	1000	1	4.81E-05	2.66E-02	0.58	29	2.9		
10:5779001	10	5779001	5780000	1000	1	5.85E-05	2.78E-02	-1.03	3	0.3		
10:15023001	10	15023001	15024000	1000	1	7.10E-05	2.97E-02	-0.743	10	1		
10:16921001	10	16921001	16922000	1000	1	4.69E-05	2.66E-02	-0.649	8	0.8		
10:21801001	10	21801001	21802000	1000	1	3.34E-05	2.39E-02	-0.845	4	0.4		
10:22418001	10	22418001	22419000	1000	1	1.80E-06	8.13E-03	-1.314	3	0.3		
10:22505001	10	22505001	22508000	3000	1	7.86E-05	3.09E-02	-0.734	25	0.83		
10:25190001	10	25190001	25193000	3000	1	3.60E-05	2.41E-02	-1.25	19	0.63	DNAJC1	Transcription
10:28838001	10	28838001	28839000	1000	1	3.50E-05	2.39E-02	-0.803	10	1		
10:31543001	10	31543001	31544000	1000	1	3.92E-05	2.49E-02	-1.335	8	0.8	LOC122448461	
10:44224001	10	44224001	44225000	1000	1	7.01E-05	2.95E-02	0.73	29	2.9	ENTPD6	Signaling
10:46713001	10	46713001	46715000	2000	1	2.79E-05	2.21E-02	0.663	66	3.3	PITRM1;LOC122447904	Protease
10:59859001	10	59859001	59860000	1000	1	7.34E-06	1.22E-02	-0.738	9	0.9	DEFB121	Signaling
10:62839001	10	62839001	62841000	2000	1	6.35E-05	2.86E-02	-0.728	13	0.65	PIGU	
10:63137001	10	63137001	63138000	1000	1	2.19E-05	2.05E-02	0.59	24	2.4	MYH7B	
10:71377001	10	71377001	71378000	1000	1	7.38E-05	3.00E-02	-0.67	19	1.9	LOC122449098;LOC122448507;LOC122449022	
10:72643001	10	72643001	72644000	1000	1	6.67E-05	2.90E-02	-1.031	4	0.4	LOC122448936	
11:1667001	11	1667001	1669000	2000	1	1.78E-05	1.89E-02	-0.837	11	0.55		
11:7240001	11	7240001	7242000	2000	1	9.57E-05	3.21E-02	-0.861	12	0.6	CNTN5	
11:8008001	11	8008001	8010000	2000	1	1.14E-05	1.56E-02	-0.853	10	0.5	CNTN5	
11:10925001	11	10925001	10927000	2000	1	3.52E-05	2.39E-02	-0.809	28	1.4		
11:15303001	11	15303001	15305000	2000	1	3.30E-05	2.39E-02	-1.022	6	0.3	CUL5	Proteolysis
11:51721001	11	51721001	51722000	1000	1	4.46E-05	2.62E-02	0.648	8	0.8	MYO7A;LOC122449862;GDPD4	Cytoskeleton;Signaling
11:52446001	11	52446001	52448000	2000	1	2.71E-06	8.36E-03	-0.728	33	1.65	SLC5A12	Transport
11:52718001	11	52718001	52720000	2000	1	9.29E-05	3.20E-02	-1.005	9	0.45	FIBIN	
11:70732001	11	70732001	70733000	1000	1	6.88E-05	2.92E-02	0.574	27	2.7	F2;LOC122449816;CKAP5	Protease;Cytoskeleton
11:71267001	11	71267001	71268000	1000	1	6.35E-05	2.86E-02	0.647	23	2.3	LOC122449474;SLC39A13	Transport
11:76986001	11	76986001	76989000	3000	2	7.27E-05	2.98E-02	0.645	147	4.9	LOC122449527	
11:77982001	11	77982001	77985000	3000	1	3.48E-05	2.39E-02	0.815	80	2.67		
12:1479001	12	1479001	1481000	2000	1	9.27E-05	3.20E-02	-0.967	10	0.5		
12:7385001	12	7385001	7387000	2000	1	6.80E-05	2.91E-02	0.744	112	5.6	TRAPPC9	
12:9025001	12	9025001	9026000	1000	1	8.44E-05	3.15E-02	0.755	23	2.3	ADGRB1;LOC122451499	Signaling
12:10357001	12	10357001	10358000	1000	1	2.73E-05	2.20E-02	0.737	11	1.1	SCRT1;TMEM249;FBXL6;SLC52A2	Transcription;Transport
12:11916001	12	11916001	11918000	2000	1	1.66E-05	1.82E-02	-1.26	7	0.35		
12:13815001	12	13815001	13816000	1000	1	8.00E-05	3.12E-02	0.598	26	2.6	MTSS1	Cytoskeleton
12:20829001	12	20829001	20832000	3000	1	4.64E-05	2.66E-02	-0.797	21	0.7		
12:36770001	12	36770001	36772000	2000	1	5.30E-05	2.71E-02	-0.671	10	0.5		
12:38881001	12	38881001	38882000	1000	1	5.25E-05	2.69E-02	-0.979	0	0		
12:47494001	12	47494001	47495000	1000	1	2.07E-06	8.13E-03	-0.933	4	0.4		
12:67300001	12	67300001	67302000	2000	1	3.04E-05	2.32E-02	-1.01	12	0.6		
12:69252001	12	69252001	69253000	1000	1	6.03E-05	2.80E-02	-0.625	10	1	SLC26A7	Transport
13:63001	13	63001	64000	1000	1	5.38E-05	2.71E-02	0.742	26	2.6	TNNT2;LAD1	Cytoskeleton;Development
13:341001	13	341001	346000	5000	1	5.05E-05	2.66E-02	0.703	309	6.18	NAV1	
13:549001	13	549001	550000	1000	1	8.16E-07	5.29E-03	0.801	30	3	GPR37L1;LOC122452023;ARL8A	Signaling
13:21123001	13	21123001	21124000	1000	1	6.82E-05	2.91E-02	-0.599	20	2		
13:26025001	13	26025001	26026000	1000	1	1.55E-06	7.70E-03	-1.028	2	0.2		
13:42027001	13	42027001	42028000	1000	1	8.69E-06	1.41E-02	-0.805	4	0.4		
13:48369001	13	48369001	48370000	1000	1	9.85E-06	1.47E-02	-0.946	7	0.7	LOC122451656	
13:50838001	13	50838001	50839000	1000	1	7.50E-05	3.03E-02	-0.66	17	1.7	DNAH14	Cytoskeleton
13:52906001	13	52906001	52908000	2000	1	3.34E-05	2.39E-02	-0.705	18	0.9	TAF1A;TRNAG-UCC	
13:59514001	13	59514001	59516000	2000	1	6.17E-05	2.82E-02	-0.897	13	0.65	USH2A	Extracellular Matrix
14:11443001	14	11443001	11445000	2000	1	9.47E-06	1.45E-02	0.719	49	2.45	SHB	

14:24135001	14	24135001	24136000	1000	1	9.40E-05	3.21E-02	-0.99	4	0.4	GDA;LOC122453162	Metabolism
14:24208001	14	24208001	24210000	2000	1	1.93E-07	3.25E-03	-1.128	6	0.3		
14:30156001	14	30156001	30157000	1000	1	1.07E-09	2.09E-04	-1.218	1	0.1		
14:38030001	14	38030001	38032000	2000	1	5.37E-05	2.71E-02	-1.109	6	0.3		
14:41051001	14	41051001	41052000	1000	1	7.93E-05	3.11E-02	-0.853	5	0.5		
14:45065001	14	45065001	45066000	1000	1	2.69E-05	2.18E-02	-0.804	6	0.6		
14:49038001	14	49038001	49039000	1000	1	3.66E-05	2.42E-02	-0.987	6	0.6	LOC122452871	
14:64202001	14	64202001	64211000	9000	1	5.63E-05	2.73E-02	-0.751	140	1.56		
14:64303001	14	64303001	64315000	12000	1	5.49E-05	2.71E-02	-0.711	198	1.65		
14:65781001	14	65781001	65791000	10000	1	7.12E-05	2.97E-02	-0.819	142	1.42		
14:65793001	14	65793001	65817000	24000	1	8.93E-05	3.16E-02	-0.719	353	1.47		
14:65850001	14	65850001	65875000	25000	1	7.21E-05	2.98E-02	-0.736	360	1.44		
14:68006001	14	68006001	68010000	4000	3	5.82E-06	1.11E-02	-1.073	4	0.1		
14:69003001	14	69003001	69016000	13000	1	8.47E-05	3.15E-02	-0.732	202	1.55		
14:74288001	14	74288001	74305000	17000	2	3.45E-05	2.39E-02	-0.787	266	1.56		
14:74315001	14	74315001	74319000	4000	1	4.37E-06	9.92E-03	-0.712	69	1.73		
15:514001	15	514001	515000	1000	1	2.38E-05	2.11E-02	-0.912	15	1.5		
15:3778001	15	3778001	3779000	1000	1	5.45E-06	1.07E-02	-1.05	12	1.2		
15:9336001	15	9336001	9338000	2000	1	2.13E-05	2.03E-02	-0.993	8	0.4		
15:23611001	15	23611001	23613000	2000	1	5.10E-05	2.67E-02	-0.968	4	0.2		
15:41019001	15	41019001	41021000	2000	1	8.15E-05	3.13E-02	-0.697	16	0.8		
15:43302001	15	43302001	43304000	2000	1	6.68E-05	2.90E-02	-0.936	10	0.5		
15:48958001	15	48958001	48960000	2000	1	5.69E-05	2.75E-02	-1.049	6	0.3	SPC25;G6PC2	Signaling
15:63342001	15	63342001	63343000	1000	1	1.24E-06	6.82E-03	-0.959	5	0.5		
15:67930001	15	67930001	67931000	1000	1	7.49E-05	3.03E-02	-0.628	31	3.1		
15:74204001	15	74204001	74205000	1000	1	1.54E-06	7.70E-03	0.781	48	4.8	LIMS2;GPR17;MYO7B	Cytoskeleton;Signaling;Cytoskeleton
16:8188001	16	8188001	8189000	1000	1	4.45E-06	9.92E-03	-1.13	4	0.4		
16:11591001	16	11591001	11594000	3000	2	4.94E-07	4.23E-03	-1.059	11	0.37		
16:18023001	16	18023001	18024000	1000	1	5.21E-07	4.23E-03	-1.036	4	0.4	PDE4D	Signaling
16:38726001	16	38726001	38727000	1000	1	4.39E-05	2.60E-02	-0.971	4	0.4	LOC122454260	
16:38770001	16	38770001	38773000	3000	1	1.01E-05	1.48E-02	-1.21	15	0.5		
16:47757001	16	47757001	47758000	1000	1	2.26E-05	2.08E-02	-1.024	3	0.3		
16:48354001	16	48354001	48355000	1000	1	7.37E-06	1.22E-02	-1.142	6	0.6	CDH12	Cytoskeleton
16:49369001	16	49369001	49370000	1000	1	5.15E-05	2.67E-02	-0.917	3	0.3		
16:49836001	16	49836001	49837000	1000	1	5.73E-05	2.75E-02	-0.961	6	0.6	CDH18	Cytoskeleton
17:814001	17	814001	815000	1000	1	3.08E-05	2.34E-02	-0.927	2	0.2		
17:3067001	17	3067001	3069000	2000	1	4.54E-07	4.21E-03	0.821	63	3.15	EVL	Cytoskeleton
17:3234001	17	3234001	3235000	1000	1	6.39E-05	2.86E-02	0.745	32	3.2	SLC25A47;WARS1	Transport;Translation
17:4150001	17	4150001	4151000	1000	1	9.11E-05	3.19E-02	-0.77	6	0.6		
17:6235001	17	6235001	6237000	2000	1	5.01E-06	1.05E-02	0.831	99	4.95	KLC1;XRCC3;ZFYVE21	Cytoskeleton;Transcription
17:6622001	17	6622001	6624000	2000	1	2.21E-05	2.05E-02	0.95	53	2.65	LOC122454781	
17:7068001	17	7068001	7071000	3000	1	9.36E-05	3.21E-02	0.831	159	5.3	INF2;ADSS1	Metabolism
17:7981001	17	7981001	7983000	2000	1	5.41E-05	2.71E-02	-0.958	11	0.55	LOC122419713	
17:13231001	17	13231001	13233000	2000	1	6.39E-05	2.86E-02	-0.928	5	0.25	CATSPERB;LOC122454884	Transport
17:17538001	17	17538001	17539000	1000	1	8.79E-06	1.41E-02	-1.207	4	0.4		
17:27669001	17	27669001	27670000	1000	1	2.12E-05	2.03E-02	-0.754	6	0.6	LOC122454918	
17:36860001	17	36860001	36861000	1000	1	8.40E-05	3.15E-02	0.734	22	2.2	TMEM266	
17:39847001	17	39847001	39849000	2000	1	1.67E-05	1.82E-02	-0.947	8	0.4	FAM189A1	
17:41139001	17	41139001	41140000	1000	1	4.62E-05	2.66E-02	-0.736	6	0.6	IL16	Cytokine
18:7868001	18	7868001	7869000	1000	1	3.38E-05	2.39E-02	-0.861	3	0.3		
18:8322001	18	8322001	8323000	1000	1	9.87E-05	3.27E-02	-0.705	11	1.1	LOC122420636;LOC122420587	
18:9804001	18	9804001	9805000	1000	1	3.65E-05	2.42E-02	-0.824	1	0.1	LOC122420399;LOC122420482	
18:13496001	18	13496001	13498000	2000	1	2.88E-06	8.54E-03	-0.857	9	0.45	NCR1	Immune
18:13518001	18	13518001	13519000	1000	1	1.14E-05	1.56E-02	-0.856	10	1	NCR1;LOC122420990	Immune
18:14046001	18	14046001	14049000	3000	1	4.19E-06	9.81E-03	0.84	149	4.97	FIZ1;ZNF524;ZNF865;ZNF784	Transcription
18:14780001	18	14780001	14781000	1000	1	4.17E-05	2.55E-02	-0.938	3	0.3	LOC122420188;LOC122420173	
18:16109001	18	16109001	16110000	1000	1	2.29E-05	2.09E-02	0.629	32	3.2	ARHGEF1;CD79A	Transcription;Immune
18:17340001	18	17340001	17342000	2000	1	6.72E-06	1.18E-02	0.698	39	1.95	PVR;LOC122420740	
18:18692001	18	18692001	18694000	2000	1	2.67E-05	2.18E-02	0.773	65	3.25	PRKD2	Signaling
18:18720001	18	18720001	18723000	3000	1	7.88E-05	3.09E-02	0.775	116	3.87	PRKD2;STRN4	Signaling
18:19518001	18	19518001	19523000	5000	1	5.57E-05	2.71E-02	0.681	211	4.22	EHD2;NOP53;LOC122421844;TRNAM-CAU	Transport
18:20593001	18	20593001	20595000	2000	1	8.63E-06	1.41E-02	0.765	69	3.45	PPFIA3;HRC;TRPM4	Transport
18:21594001	18	21594001	21596000	2000	1	3.67E-05	2.42E-02	0.707	56	2.8	LRRC4B;LOC122421264	
18:22461001	18	22461001	22462000	1000	1	6.29E-05	2.86E-02	0.706	21	2.1	B9D2;TGFB1	Development;Growth Factors
18:23377001	18	23377001	23379000	2000	1	9.87E-05	3.27E-02	0.588	52	2.6	AKT2;LOC122420501	Signaling
18:26505001	18	26505001	26511000	6000	1	1.00E-05	1.48E-02	0.689	174	2.9	KMT2B;ZBTB32	Epigenetic;Transcription
18:51448001	18	51448001	51449000	1000	1	1.81E-05	1.89E-02	0.902	43	4.3	ZFPM1	Transcription
18:53435001	18	53435001	53436000	1000	1	8.68E-05	3.15E-02	0.819	28	2.8	C18H16orf74	
18:54521001	18	54521001	54523000	2000	1	1.85E-05	1.92E-02	-0.881	13	0.65	ATP2C2;TRNAE-UUC	Transport
18:56820001	18	56820001	56823000	3000	1	7.25E-08	1.76E-03	0.875	85	2.83	LOC122421712;LOC122421713	
19:1660001	19	1660001	1661000	1000	1	9.44E-05	3.21E-02	-0.609	12	1.2		
19:35416001	19	35416001	35417000	1000	1	3.57E-05	2.39E-02	-0.757	4	0.4		

19:41605001	19	41605001	41606000	1000	1	8.55E-05	3.15E-02	-0.968	5	0.5	MANBA	Golgi
19:54755001	19	54755001	54756000	1000	1	2.52E-05	2.17E-02	-0.97	2	0.2		
19:59360001	19	59360001	59362000	2000	1	1.04E-07	2.24E-03	-0.957	9	0.45		
20:11617001	20	11617001	11618000	1000	1	7.79E-05	3.07E-02	-0.695	8	0.8		
20:15664001	20	15664001	15665000	1000	1	6.10E-05	2.82E-02	-0.934	5	0.5		
20:22416001	20	22416001	22429000	13000	1	8.25E-05	3.13E-02	-1.386	145	1.12	LOC122422614	
20:22456001	20	22456001	22457000	1000	1	4.63E-06	9.92E-03	-1.711	11	1.1	LOC122422614	
20:22506001	20	22506001	22510000	4000	1	2.61E-05	2.18E-02	-1.5	62	1.55	LOC122422614	
20:22542001	20	22542001	22548000	6000	1	9.08E-05	3.19E-02	-1.366	103	1.72	LOC122422614;LOC122422694	
20:22550001	20	22550001	22559000	9000	2	3.26E-06	9.07E-03	-1.498	89	0.99	LOC122422614;LOC122422694	
20:22578001	20	22578001	22580000	2000	1	5.09E-05	2.67E-02	-1.47	20	1	LOC122422694	
20:22598001	20	22598001	22603000	5000	1	3.89E-05	2.49E-02	-1.364	96	1.92	LOC122422694	
20:22638001	20	22638001	22641000	3000	1	4.71E-05	2.66E-02	-1.66	41	1.37		
20:22652001	20	22652001	22656000	4000	2	3.43E-05	2.39E-02	-1.576	36	0.9		
20:22657001	20	22657001	22658000	1000	1	9.11E-05	3.19E-02	-1.521	10	1		
20:22682001	20	22682001	22686000	4000	1	2.65E-06	8.36E-03	-1.979	32	0.8		
20:22741001	20	22741001	22742000	1000	1	5.70E-05	2.75E-02	-1.189	9	0.9	LOC122422605	
20:22815001	20	22815001	22821000	6000	1	4.10E-05	2.55E-02	-1.07	104	1.73	LOC122422607	
20:22823001	20	22823001	22832000	9000	1	6.71E-05	2.91E-02	-1.427	86	0.96	LOC122422607	
20:22893001	20	22893001	22900000	7000	1	4.78E-05	2.66E-02	-1.384	167	2.39	LOC122422610;LOC122422608	
20:22901001	20	22901001	22908000	7000	3	3.48E-06	9.28E-03	-1.786	65	0.93	LOC122422610;LOC122422608	
20:22911001	20	22911001	22913000	2000	1	5.12E-05	2.67E-02	-1.155	19	0.95	LOC122422610;LOC122422613	
20:22927001	20	22927001	22943000	16000	2	1.89E-05	1.93E-02	-1.846	152	0.95	LOC122422610;LOC122422613	
20:22972001	20	22972001	22974000	2000	1	9.63E-06	1.46E-02	-1.571	7	0.35	LOC122422610;LOC122422604	
20:22975001	20	22975001	22987000	12000	1	4.04E-05	2.53E-02	-1.527	114	0.95	LOC122422610;LOC122422604	
20:23000001	20	23000001	23003000	3000	1	8.26E-05	3.13E-02	-1.177	18	0.6	LOC122422610;LOC122422699	
20:30611001	20	30611001	30612000	1000	1	2.43E-06	8.13E-03	-1.114	3	0.3		
20:46030001	20	46030001	46032000	2000	1	2.29E-05	2.09E-02	-0.736	10	0.5		
20:54116001	20	54116001	54118000	2000	1	2.03E-05	2.01E-02	0.673	37	1.85	LOC122422752	
21:77001	21	77001	79000	2000	1	6.94E-05	2.93E-02	0.658	41	2.05	ACR;SHANK3	Protease
21:130001	21	130001	131000	1000	1	6.42E-05	2.87E-02	0.645	25	2.5	SHANK3	
21:295001	21	295001	297000	2000	1	4.59E-05	2.66E-02	0.691	62	3.1	MIOX;ADM2;SBF1	Metabolism;Hormone;Signaling
21:430001	21	430001	433000	3000	1	1.36E-05	1.71E-02	1.007	170	5.67	PLXNB2	
21:857001	21	857001	860000	3000	1	5.57E-06	1.07E-02	0.975	162	5.4	BRD1	Transcription
21:1243001	21	1243001	1244000	1000	1	6.55E-06	1.18E-02	0.713	27	2.7		
21:2661001	21	2661001	2662000	1000	1	5.29E-06	1.06E-02	0.775	33	3.3		
21:3653001	21	3653001	3654000	1000	1	3.27E-07	3.89E-03	0.862	27	2.7	LOC122424035	
21:4632001	21	4632001	4635000	3000	1	7.35E-05	2.99E-02	0.592	87	2.9	PRR5	
21:5224001	21	5224001	5225000	1000	1	2.87E-05	2.25E-02	0.815	25	2.5	PNPLA3	
21:5836001	21	5836001	5838000	2000	1	5.43E-05	2.71E-02	0.765	94	4.7	SCUBE1	Extracellular Matrix
21:5869001	21	5869001	5873000	4000	2	4.16E-08	1.51E-03	0.985	162	4.05	SCUBE1	Extracellular Matrix
21:5918001	21	5918001	5921000	3000	1	1.23E-05	1.60E-02	0.804	139	4.63	TTLL12	
21:9762001	21	9762001	9764000	2000	1	4.18E-06	9.81E-03	0.708	54	2.7	SLC16A8;PICK1;LOC122423074	Transport;Transport
21:10405001	21	10405001	10406000	1000	1	6.01E-05	2.80E-02	0.773	40	4	MICAL3	
21:10438001	21	10438001	10439000	1000	1	2.93E-06	8.54E-03	0.731	37	3.7	MICAL3;BID	
21:18916001	21	18916001	18917000	1000	1	1.62E-05	1.82E-02	-0.759	5	0.5	LOC122423893;LOC122423894	
21:19901001	21	19901001	19902000	1000	1	6.61E-05	2.90E-02	-1.04	3	0.3	LOC122423609	
21:37527001	21	37527001	37528000	1000	1	9.49E-05	3.21E-02	-0.754	5	0.5		
21:42317001	21	42317001	42319000	2000	1	4.71E-05	2.66E-02	-1.345	36	1.8		
21:45776001	21	45776001	45778000	2000	1	5.97E-05	2.80E-02	-0.651	7	0.35	LOC122423505	
21:50327001	21	50327001	50328000	1000	1	1.54E-05	1.81E-02	-0.777	4	0.4		
21:54464001	21	54464001	54465000	1000	1	1.99E-06	8.13E-03	0.672	15	1.5	BTBD11	Cytoskeleton
21:59257001	21	59257001	59258000	1000	1	1.42E-06	7.44E-03	0.787	9	0.9	IL2RB	Receptor
21:59358001	21	59358001	59360000	2000	1	3.55E-05	2.39E-02	0.789	38	1.9	RAC2	Signaling
22:691001	22	691001	692000	1000	1	8.72E-05	3.15E-02	0.772	26	2.6		
22:3977001	22	3977001	3978000	1000	1	6.11E-05	2.82E-02	0.702	42	4.2	LOC122424673	
22:10024001	22	10024001	10029000	5000	1	8.64E-05	3.15E-02	0.829	211	4.22	BSN	
22:13884001	22	13884001	13886000	2000	1	8.12E-05	3.13E-02	-0.829	9	0.45	CACNA2D3	Transport
22:19375001	22	19375001	19376000	1000	1	5.19E-05	2.67E-02	-0.77	4	0.4	FHIT	Signaling
22:44417001	22	44417001	44418000	1000	1	4.83E-05	2.66E-02	0.585	14	1.4	GASK1A;LOC122425004;HIGD1A;CCDC13	
23:2492001	23	2492001	2493000	1000	1	2.34E-06	8.13E-03	-0.806	5	0.5	LOC122425960;KATNAL2	Cytoskeleton
23:7479001	23	7479001	7481000	2000	1	3.46E-07	3.89E-03	-0.935	12	0.6	DCC	
23:16959001	23	16959001	16960000	1000	1	7.64E-05	3.04E-02	-1.079	4	0.4	LOC122425698;LOC122425696	
23:17463001	23	17463001	17464000	1000	1	1.12E-06	6.39E-03	0.799	25	2.5	RBFA;LOC122425780;LOC122425772	
23:17659001	23	17659001	17660000	1000	1	3.92E-06	9.64E-03	0.909	51	5.1	CTDP1	Signaling
23:17759001	23	17759001	17760000	1000	1	3.68E-06	9.41E-03	0.951	49	4.9	NFATC1	Transcription
23:43642001	23	43642001	43644000	2000	1	3.73E-06	9.41E-03	-0.815	13	0.65	CDH2	Cytoskeleton
23:44588001	23	44588001	44589000	1000	1	4.46E-05	2.62E-02	-0.803	7	0.7	CHST9	Transport
23:48429001	23	48429001	48430000	1000	1	9.18E-05	3.20E-02	-0.913	0	0		
23:52342001	23	52342001	52343000	1000	1	8.00E-07	5.29E-03	-0.87	6	0.6	DLGAP1	Cytoskeleton

24:26855001	24	26855001	26856000	1000	1	2.69E-06	8.36E-03	-0.881	19	1.9	LOC122426328	
24:27318001	24	27318001	27319000	1000	1	2.47E-05	2.16E-02	0.723	16	1.6	OBSL1;TMEM198;CHPF	Golgi
24:35788001	24	35788001	35789000	1000	1	8.48E-05	3.15E-02	-0.684	4	0.4		
24:44280001	24	44280001	44281000	1000	1	4.38E-05	2.60E-02	-0.681	9	0.9	CFLAR	Protease
24:49992001	24	49992001	49993000	1000	1	6.58E-05	2.90E-02	-0.921	5	0.5		
24:51375001	24	51375001	51376000	1000	1	8.65E-05	3.15E-02	-0.951	2	0.2		
24:52993001	24	52993001	52994000	1000	1	3.72E-05	2.42E-02	-0.875	4	0.4		
25:1692001	25	1692001	1694000	2000	1	8.92E-05	3.16E-02	0.91	74	3.7	LRP1	Binding Proteins
25:1708001	25	1708001	1709000	1000	1	4.65E-06	9.92E-03	0.923	78	7.8	LRP1	Binding Proteins
25:2077001	25	2077001	2079000	2000	1	3.24E-05	2.38E-02	0.717	50	2.5	ARHGEF25;LOC122427336;LOC122427340;B4GALNT1	Transcription
25:7005001	25	7005001	7006000	1000	1	6.74E-05	2.91E-02	-1.275	2	0.2		
25:20986001	25	20986001	20987000	1000	1	7.29E-05	2.98E-02	-0.606	6	0.6		
25:29721001	25	29721001	29722000	1000	1	2.52E-05	2.17E-02	0.627	33	3.3	HOXC10;HOXC8;LOC122427283;HOXC9;LOC122427285	Development
25:29978001	25	29978001	29979000	1000	1	4.69E-05	2.66E-02	0.616	24	2.4	HNRNPA1;NFE2	Transcription
25:30457001	25	30457001	30459000	2000	1	4.91E-05	2.66E-02	-1.035	14	0.7	LOC122427759	
25:41039001	25	41039001	41041000	2000	1	5.01E-05	2.66E-02	-0.795	8	0.4		
26:4802001	26	4802001	4803000	1000	1	1.79E-05	1.89E-02	-1.032	4	0.4	CRACD	
26:6914001	26	6914001	6916000	2000	1	1.58E-05	1.81E-02	-1.04	12	0.6		
26:8230001	26	8230001	8231000	1000	1	7.81E-07	5.29E-03	-1.073	5	0.5		
26:8983001	26	8983001	8984000	1000	1	5.94E-05	2.79E-02	-0.77	5	0.5		
26:16555001	26	16555001	16558000	3000	1	8.22E-05	3.13E-02	-0.926	32	1.07		
26:18199001	26	18199001	18200000	1000	1	6.93E-06	1.18E-02	-0.75	2	0.2		
26:24655001	26	24655001	24656000	1000	1	5.94E-05	2.79E-02	-0.833	1	0.1	ADAMTS3	Protease
26:37892001	26	37892001	37893000	1000	1	9.22E-05	3.20E-02	-0.762	4	0.4	PTPN13	
26:52306001	26	52306001	52308000	2000	1	7.99E-05	3.12E-02	0.8	106	5.3	UVSSA	
26:52420001	26	52420001	52421000	1000	1	5.12E-06	1.06E-02	0.906	99	9.9	CTBP1	Transcription
27:2372001	27	2372001	2373000	1000	1	4.37E-05	2.60E-02	0.788	34	3.4	URB1	
27:4486001	27	4486001	4488000	2000	1	9.27E-05	3.20E-02	-1.071	14	0.7	LOC122428966	
27:5859001	27	5859001	5860000	1000	1	9.22E-06	1.45E-02	-0.846	7	0.7		
27:10138001	27	10138001	10139000	1000	1	3.81E-07	3.89E-03	-1.049	6	0.6		
27:19311001	27	19311001	19312000	1000	1	2.22E-07	3.25E-03	-1.007	6	0.6	USP25	Protease
27:21624001	27	21624001	21625000	1000	1	2.59E-05	2.18E-02	-0.715	6	0.6	LOC122428617	
27:24419001	27	24419001	24420000	1000	1	3.70E-05	2.42E-02	-0.865	3	0.3		
27:42422001	27	42422001	42424000	2000	1	7.64E-05	3.04E-02	-0.862	12	0.6	ZPLD1	Receptor
27:46742001	27	46742001	46744000	2000	1	5.22E-07	4.23E-03	-1.014	3	0.15		
27:50168001	27	50168001	50169000	1000	1	6.83E-05	2.91E-02	-0.648	11	1.1		
28:774001	28	774001	776000	2000	2	6.68E-06	1.18E-02	0.944	122	6.1	FOXC1	
28:1180001	28	1180001	1182000	2000	1	9.66E-05	3.23E-02	0.72	114	5.7	GMDS	Metabolism
28:24813001	28	24813001	24814000	1000	1	8.48E-05	3.15E-02	-0.882	4	0.4		
28:31060001	28	31060001	31061000	1000	1	9.86E-07	5.81E-03	-1.451	4	0.4	LOC122430107	
28:31257001	28	31257001	31259000	2000	2	6.46E-05	2.87E-02	-1.254	16	0.8	LOC122429269	
28:31385001	28	31385001	31391000	6000	1	6.66E-05	2.90E-02	-1.237	73	1.22	LOC122429464	
28:31662001	28	31662001	31663000	1000	1	8.32E-05	3.13E-02	-1.026	4	0.4	LOC122429462;LOC122429474	
28:31703001	28	31703001	31704000	1000	1	5.53E-05	2.71E-02	-1.255	9	0.9	LOC122429462;LOC122429479;LOC122429471	
28:32428001	28	32428001	32430000	2000	1	5.37E-05	2.71E-02	-0.742	8	0.4	KHDRBS2	Translation
28:32950001	28	32950001	32952000	2000	1	8.75E-05	3.15E-02	-0.903	13	0.65		
28:38916001	28	38916001	38925000	9000	3	1.64E-05	1.82E-02	0.785	346	3.84	COL11A2	Extracellular Matrix
29:15859001	29	15859001	15861000	2000	1	3.03E-06	8.54E-03	-0.992	11	0.55	LOC122431103	
29:25162001	29	25162001	25165000	3000	1	2.39E-06	8.13E-03	-0.823	22	0.73	LOC122431034	
29:37769001	29	37769001	37771000	2000	1	3.48E-05	2.39E-02	0.834	65	3.25	DAGLA	Metabolism
29:42703001	29	42703001	42704000	1000	1	3.74E-05	2.42E-02	0.95	53	5.3	LOC122430821;CARNS1;RPS6KB2;LOC122430963	Golgi
29:44605001	29	44605001	44610000	5000	1	4.87E-05	2.66E-02	0.832	200	4	MUC2	Extracellular Matrix
29:44873001	29	44873001	44874000	1000	1	2.83E-05	2.23E-02	0.71	37	3.7	BRSK2	
29:45528001	29	45528001	45530000	2000	1	6.35E-05	2.86E-02	0.711	100	5	IGF2;INS;TH	Growth Factors
29:45980001	29	45980001	45981000	1000	1	5.92E-05	2.79E-02	0.596	16	1.6	KCNQ1;LOC122431064	Transport
29:46140001	29	46140001	46142000	2000	1	3.11E-05	2.35E-02	0.634	88	4.4	KCNQ1	Transport
29:46288001	29	46288001	46290000	2000	1	3.87E-05	2.48E-02	0.915	155	7.75	CARS1	Translation
29:46421001	29	46421001	46424000	3000	1	1.90E-05	1.93E-02	0.71	98	3.27	OSBPL5	
30:5983001	30	5983001	5984000	1000	1	1.77E-05	1.89E-02	-0.903	4	0.4		
30:6493001	30	6493001	6494000	1000	1	3.28E-05	2.39E-02	-0.841	7	0.7	KCTD9;CDCA2	
30:9737001	30	9737001	9738000	1000	1	8.14E-05	3.13E-02	-0.948	8	0.8	LOC122431941;LOC122431942	
30:11963001	30	11963001	11964000	1000	1	3.17E-05	2.36E-02	-0.848	3	0.3		
30:20570001	30	20570001	20571000	1000	1	6.63E-06	1.18E-02	-0.968	5	0.5		
30:22467001	30	22467001	22468000	1000	1	1.58E-05	1.81E-02	0.786	21	2.1	LOC122431747;NXNL2	Metabolism
30:22676001	30	22676001	22677000	1000	1	3.45E-05	2.39E-02	-0.902	9	0.9		
30:25552001	30	25552001	25554000	2000	1	2.55E-05	2.17E-02	-0.636	11	0.55		
30:35402001	30	35402001	35403000	1000	1	9.53E-05	3.21E-02	0.735	17	1.7	COL27A1	Extracellular Matrix
30:41470001	30	41470001	41471000	1000	1	3.50E-05	2.39E-02	0.702	49	4.9	CDK5RAP2	

30:42636001	30	42636001	42639000	3000	1	6.84E-05	2.91E-02	0.668	136	4.53	EIPR1	
31:591001	31	591001	592000	1000	1	9.94E-05	3.28E-02	-0.816	4	0.4		
31:2223001	31	2223001	2224000	1000	1	2.26E-05	2.08E-02	-0.961	5	0.5	CSMD1	
31:23260001	31	23260001	23261000	1000	1	5.22E-06	1.06E-02	-0.867	11	1.1		
31:25163001	31	25163001	25164000	1000	1	4.44E-05	2.62E-02	0.759	31	3.1	PURG;WRN;LOC122432627	Transcription;Epigenetic
31:32648001	31	32648001	32649000	1000	1	8.00E-05	3.12E-02	-0.817	1	0.1	LOC122432573	
31:38623001	31	38623001	38625000	2000	1	4.55E-06	9.92E-03	-0.963	6	0.3		
31:42698001	31	42698001	42700000	2000	1	1.18E-05	1.56E-02	0.734	32	1.6		
32:7001	32	7001	10000	3000	1	3.81E-07	3.89E-03	-1.578	30	1		
32:24001	32	24001	25000	1000	1	6.05E-05	2.80E-02	-1.337	5	0.5	LOC122433607	
32:6590001	32	6590001	6591000	1000	1	7.62E-05	3.04E-02	0.613	24	2.4	ABAT	Metabolism
32:7168001	32	7168001	7169000	1000	1	6.05E-05	2.80E-02	-0.913	1	0.1		
32:12781001	32	12781001	12782000	1000	1	6.78E-05	2.91E-02	-1.046	4	0.4	COQ7	
32:15159001	32	15159001	15160000	1000	1	9.68E-05	3.23E-02	-1.001	7	0.7	LOC122433545	
32:15161001	32	15161001	15164000	3000	1	9.56E-05	3.21E-02	-0.716	25	0.83	LOC122433545	
32:16137001	32	16137001	16139000	2000	1	2.65E-06	8.36E-03	-0.974	18	0.9		
32:20112001	32	20112001	20113000	1000	1	9.78E-05	3.26E-02	0.624	17	1.7	HIRIP3;TAOK2;TMEM219	Signaling
32:28334001	32	28334001	28337000	3000	1	7.01E-06	1.18E-02	0.899	132	4.4	CUX1;TRNAG-UCC	Development
32:29129001	32	29129001	29132000	3000	1	9.01E-05	3.18E-02	0.673	61	2.03	VGF;AP1S1;LOC122433302	Signaling;Transport
32:29447001	32	29447001	29449000	2000	1	6.64E-05	2.90E-02	0.784	97	4.85	POP7;GIGYF1;GNB2	Translation;Signaling
32:34151001	32	34151001	34157000	6000	1	2.20E-06	8.13E-03	0.909	340	5.67	TTYH3;LFNG	Transport;Golgi
32:34593001	32	34593001	34594000	1000	1	8.84E-05	3.15E-02	0.803	40	4	MAD1L1	
32:34791001	32	34791001	34793000	2000	2	5.48E-05	2.71E-02	0.869	124	6.2	TMEM184A;MAFK	Transport;Transcription
32:34823001	32	34823001	34825000	2000	1	9.24E-05	3.20E-02	0.838	119	5.95	LOC122433562;INTS1	
32:37196001	32	37196001	37198000	2000	1	6.56E-05	2.90E-02	-0.712	15	0.75	LOC122433398;LOC122433278	
32:38060001	32	38060001	38061000	1000	1	1.85E-05	1.92E-02	0.716	28	2.8	ECI1;DNASE1L2;E4F1;LOC122433216	Metabolism;Transcription
32:38176001	32	38176001	38177000	1000	1	4.71E-05	2.66E-02	0.824	69	6.9	PKD1	
32:38182001	32	38182001	38183000	1000	1	4.15E-05	2.55E-02	0.83	68	6.8	PKD1;TSC2	Signaling
32:38803001	32	38803001	38804000	1000	1	2.13E-05	2.03E-02	-1.11	10	1		
32:39541001	32	39541001	39544000	3000	1	4.84E-05	2.66E-02	0.701	111	3.7	RAB11FIP3	
32:39647001	32	39647001	39648000	1000	1	9.04E-05	3.19E-02	0.794	54	5.4	RAB40C;WFIKKN1;METTL26;TRNAG-CCC	
32:40032001	32	40032001	40034000	2000	1	5.87E-05	2.78E-02	0.732	64	3.2	TRNAR-CCG;CACNA1H	Transport
33:7774001	33	7774001	7776000	2000	1	6.85E-06	1.18E-02	-0.961	14	0.7		
X:465001	X	465001	468000	3000	1	6.29E-06	1.15E-02	-1.937	17	0.57		
X:570001	X	570001	573000	3000	1	6.80E-05	2.91E-02	-0.997	20	0.67		
X:2321001	X	2321001	2323000	2000	1	7.57E-05	3.03E-02	-1.026	4	0.2	LOC122434309	
X:15290001	X	15290001	15291000	1000	1	1.44E-05	1.77E-02	-1.05	2	0.2	LOC122435357	
X:16505001	X	16505001	16507000	2000	1	8.67E-05	3.15E-02	-0.784	17	0.85		
X:16508001	X	16508001	16510000	2000	1	1.10E-05	1.56E-02	-0.948	13	0.65		
X:16712001	X	16712001	16715000	3000	1	2.21E-06	8.13E-03	-1.063	20	0.67	LOC122434737;LOC122434738	
X:22532001	X	22532001	22534000	2000	1	2.14E-06	8.13E-03	-1.227	15	0.75		
X:26825001	X	26825001	26826000	1000	1	3.38E-05	2.39E-02	-0.808	6	0.6	CXHXorf58;LOC122434864	Metabolism
X:29763001	X	29763001	29764000	1000	1	5.46E-05	2.71E-02	-0.941	3	0.3		
X:30969001	X	30969001	30970000	1000	1	3.17E-08	1.51E-03	-0.999	5	0.5		
X:31685001	X	31685001	31687000	2000	1	2.26E-06	8.13E-03	-0.971	7	0.35		
X:31721001	X	31721001	31722000	1000	1	2.47E-05	2.16E-02	-0.872	5	0.5	CYSLTR1	Signaling
X:34178001	X	34178001	34180000	2000	1	2.35E-06	8.13E-03	-0.788	12	0.6	LOC122435511	
X:35659001	X	35659001	35661000	2000	1	1.84E-06	8.13E-03	-0.745	10	0.5	LOC122436013	
X:35959001	X	35959001	35960000	1000	1	1.66E-05	1.82E-02	-0.776	8	0.8	LOC122436012	
X:36616001	X	36616001	36617000	1000	1	2.74E-05	2.20E-02	-0.893	3	0.3	PHKA1;LOC122434626	Signaling
X:36752001	X	36752001	36753000	1000	1	1.63E-06	7.89E-03	-1.009	5	0.5	HDAC8	
X:38300001	X	38300001	38304000	4000	1	6.46E-05	2.87E-02	-0.665	36	0.9	LOC122435518	
X:38460001	X	38460001	38462000	2000	1	1.11E-05	1.56E-02	-0.804	12	0.6	LOC122435523	
X:39432001	X	39432001	39433000	1000	1	4.97E-05	2.66E-02	-0.852	3	0.3		
X:47364001	X	47364001	47365000	1000	1	4.15E-05	2.55E-02	-0.76	3	0.3		
X:49131001	X	49131001	49132000	1000	1	1.12E-05	1.56E-02	-0.806	5	0.5		
X:50653001	X	50653001	50655000	2000	1	2.34E-05	2.10E-02	-0.979	11	0.55	LOC122435329	
X:50708001	X	50708001	50710000	2000	1	5.50E-06	1.07E-02	-0.873	20	1	LOC122434874	
X:52492001	X	52492001	52494000	2000	1	1.71E-06	7.90E-03	-1.009	14	0.7	DGKK	Signaling
X:52644001	X	52644001	52645000	1000	1	9.26E-05	3.20E-02	-0.865	4	0.4		
X:53702001	X	53702001	53703000	1000	1	2.21E-05	2.05E-02	-0.715	9	0.9	LOC122435015	
X:54704001	X	54704001	54705000	1000	1	5.61E-05	2.73E-02	-0.961	6	0.6	FAM120C	
X:55169001	X	55169001	55171000	2000	1	6.91E-05	2.93E-02	-0.997	11	0.55		
X:57108001	X	57108001	57109000	1000	1	4.59E-05	2.66E-02	-0.91	2	0.2	LOC122434607;LOC122434606	
X:59387001	X	59387001	59389000	2000	1	1.14E-05	1.56E-02	-1.081	10	0.5	OPHN1	Signaling
X:59515001	X	59515001	59516000	1000	1	3.17E-05	2.36E-02	-0.813	6	0.6	OPHN1	Signaling
X:60362001	X	60362001	60363000	1000	1	1.38E-05	1.72E-02	-0.834	7	0.7	LOC122434808	
X:60497001	X	60497001	60500000	3000	1	3.37E-06	9.22E-03	-0.94	48	1.6	LOC122434808;LOC122435120	
X:61213001	X	61213001	61214000	1000	1	1.13E-05	1.56E-02	-0.895	4	0.4		
X:61236001	X	61236001	61237000	1000	1	8.75E-05	3.15E-02	-0.848	4	0.4		

X:61238001	X	61238001	61239000	1000	1	5.42E-08	1.51E-03	-1.196	6	0.6		
X:61247001	X	61247001	61248000	1000	1	1.45E-05	1.77E-02	-0.864	5	0.5		
X:61717001	X	61717001	61720000	3000	1	3.38E-05	2.39E-02	-1.018	25	0.83	LOC122435576	
X:61722001	X	61722001	61724000	2000	1	1.79E-05	1.89E-02	-0.891	22	1.1	LOC122435576	
X:62295001	X	62295001	62296000	1000	1	4.17E-06	9.81E-03	-0.994	1	0.1		
X:62770001	X	62770001	62772000	2000	1	8.45E-05	3.15E-02	-0.789	10	0.5		
X:65814001	X	65814001	65816000	2000	1	6.56E-06	1.18E-02	-1.002	9	0.45	LOC122435869	
X:67628001	X	67628001	67630000	2000	1	2.36E-05	2.11E-02	-0.917	5	0.25	DMD	Proteolysis
X:69170001	X	69170001	69171000	1000	1	2.15E-05	2.04E-02	-0.726	11	1.1		
X:70064001	X	70064001	70068000	4000	2	1.38E-05	1.72E-02	-1.061	32	0.8		
X:70086001	X	70086001	70090000	4000	1	1.10E-05	1.56E-02	-1.014	39	0.98	LOC122435885	
X:77795001	X	77795001	77798000	3000	1	8.66E-05	3.15E-02	-0.865	19	0.63		
X:77963001	X	77963001	77966000	3000	1	4.71E-05	2.66E-02	-0.812	19	0.63	APOOL	
X:78251001	X	78251001	78252000	1000	1	8.25E-05	3.13E-02	-0.868	3	0.3	LOC122434305	
X:79220001	X	79220001	79221000	1000	1	3.12E-05	2.35E-02	-0.717	3	0.3		
X:79570001	X	79570001	79572000	2000	1	4.97E-05	2.66E-02	-0.613	16	0.8		
X:80445001	X	80445001	80446000	1000	1	6.23E-05	2.84E-02	-0.651	17	1.7		
X:80855001	X	80855001	80856000	1000	1	2.36E-06	8.13E-03	-0.914	2	0.2		
X:80923001	X	80923001	80925000	2000	1	8.91E-05	3.16E-02	-0.991	9	0.45		
X:81636001	X	81636001	81638000	2000	1	2.71E-05	2.19E-02	-0.824	8	0.4		
X:82052001	X	82052001	82053000	1000	1	2.90E-05	2.27E-02	-0.776	7	0.7		
X:82182001	X	82182001	82183000	1000	1	3.89E-06	9.64E-03	-1.217	1	0.1		
X:82650001	X	82650001	82652000	2000	1	2.86E-05	2.25E-02	-0.785	23	1.15		
X:82839001	X	82839001	82841000	2000	1	8.73E-09	8.48E-04	-1.063	8	0.4		
X:84687001	X	84687001	84688000	1000	1	9.83E-05	3.27E-02	-0.639	7	0.7	PCDH11X	Cytoskeleton
X:85156001	X	85156001	85158000	2000	1	7.75E-05	3.06E-02	-0.835	5	0.25		
X:85420001	X	85420001	85422000	2000	1	8.80E-05	3.15E-02	-0.793	9	0.45		
X:85492001	X	85492001	85493000	1000	1	4.33E-05	2.59E-02	-0.953	4	0.4		
X:85690001	X	85690001	85691000	1000	1	2.67E-05	2.18E-02	-0.656	4	0.4		
X:88781001	X	88781001	88784000	3000	1	4.68E-05	2.66E-02	-1.036	32	1.07		
X:89068001	X	89068001	89069000	1000	1	9.21E-06	1.45E-02	-0.983	2	0.2		
X:89276001	X	89276001	89279000	3000	1	5.54E-06	1.07E-02	-0.79	50	1.67	LOC122435645	
X:90225001	X	90225001	90226000	1000	1	8.16E-05	3.13E-02	-0.681	7	0.7		
X:90701001	X	90701001	90702000	1000	1	4.32E-06	9.92E-03	-0.926	1	0.1		
X:90743001	X	90743001	90745000	2000	1	6.93E-05	2.93E-02	-0.884	12	0.6		
X:90983001	X	90983001	90985000	2000	1	4.97E-05	2.66E-02	-0.858	12	0.6		
X:93975001	X	93975001	93976000	1000	1	3.69E-05	2.42E-02	-0.851	1	0.1	ZMAT1	
X:94154001	X	94154001	94155000	1000	1	1.49E-05	1.79E-02	-0.769	7	0.7		
X:97003001	X	97003001	97006000	3000	1	3.25E-05	2.38E-02	-0.958	20	0.67		
X:97266001	X	97266001	97268000	2000	1	2.13E-06	8.13E-03	-0.964	1	0.05		
X:97529001	X	97529001	97530000	1000	1	4.10E-05	2.55E-02	-0.991	8	0.8		
X:97589001	X	97589001	97602000	13000	2	3.02E-06	8.54E-03	-1.081	88	0.68	LOC122435661;LOC122435662	
X:97615001	X	97615001	97618000	3000	1	3.86E-05	2.48E-02	-0.756	20	0.67		
X:97636001	X	97636001	97639000	3000	1	1.99E-06	8.13E-03	-1.187	13	0.43		
X:97680001	X	97680001	97682000	2000	1	1.58E-05	1.81E-02	-0.847	13	0.65		
X:99328001	X	99328001	99331000	3000	1	7.58E-05	3.03E-02	-1.237	18	0.6	LOC122436025	
X:99600001	X	99600001	99604000	4000	1	4.83E-05	2.66E-02	-1.271	33	0.82	LOC122435824	
X:99607001	X	99607001	99609000	2000	1	9.21E-05	3.20E-02	-1.017	16	0.8	LOC122435824	
X:99617001	X	99617001	99621000	4000	1	3.63E-06	9.41E-03	-1.208	35	0.88	LOC122435824;LOC122435672;LOC122435863	
X:99625001	X	99625001	99626000	1000	1	7.16E-05	2.98E-02	-0.87	14	1.4	LOC122435824;LOC122435672;LOC122435863	
X:99671001	X	99671001	99673000	2000	1	3.52E-05	2.39E-02	-1.048	18	0.9	LOC122435863	
X:100080001	X	100080001	100081000	1000	1	9.58E-05	3.21E-02	-0.671	4	0.4	GABRA3	Ion Channel
X:100290001	X	100290001	100291000	1000	1	1.46E-05	1.77E-02	-0.926	2	0.2		
X:103121001	X	103121001	103123000	2000	1	2.21E-05	2.05E-02	-0.734	14	0.7		
X:103193001	X	103193001	103194000	1000	1	3.54E-05	2.39E-02	-0.878	2	0.2		
X:103938001	X	103938001	103939000	1000	1	2.01E-05	2.00E-02	-1.078	2	0.2		
X:104429001	X	104429001	104431000	2000	1	2.50E-06	8.24E-03	-0.853	9	0.45		
X:105782001	X	105782001	105784000	2000	1	9.44E-07	5.74E-03	-0.81	8	0.4		
X:105964001	X	105964001	105965000	1000	1	1.97E-05	1.98E-02	-0.875	13	1.3	LOC122434574;LOC122434398	
X:106179001	X	106179001	106182000	3000	1	3.39E-05	2.39E-02	-0.749	23	0.77		
X:106724001	X	106724001	106725000	1000	1	3.71E-06	9.41E-03	-0.97	1	0.1		
X:107558001	X	107558001	107560000	2000	1	4.99E-06	1.05E-02	-0.889	7	0.35		
X:107618001	X	107618001	107619000	1000	1	2.50E-05	2.17E-02	-0.986	3	0.3		
X:108569001	X	108569001	108570000	1000	1	3.90E-05	2.49E-02	-1.082	6	0.6		
X:108903001	X	108903001	108904000	1000	1	2.55E-05	2.17E-02	-0.814	6	0.6		
X:112273001	X	112273001	112274000	1000	1	9.98E-05	3.29E-02	-0.84	11	1.1		
X:118908001	X	118908001	118909000	1000	1	4.85E-08	1.51E-03	-0.955	3	0.3	ZNF280C	Transcription
X:119192001	X	119192001	119193000	1000	1	2.98E-05	2.31E-02	-0.961	3	0.3		
X:120027001	X	120027001	120028000	1000	1	1.02E-05	1.48E-02	-0.681	5	0.5		
X:122810001	X	122810001	122812000	2000	1	4.60E-06	9.92E-03	-0.882	10	0.5		
X:123055001	X	123055001	123056000	1000	1	7.70E-05	3.05E-02	-0.703	4	0.4		
X:124569001	X	124569001	124570000	1000	1	3.22E-05	2.38E-02	-0.933	5	0.5		

X:125052001	X	125052001	125054000	2000	1	2.29E-08	1.49E-03	-1.037	9	0.45		
X:125612001	X	125612001	125613000	1000	1	3.70E-05	2.42E-02	-0.882	2	0.2		
X:125963001	X	125963001	125965000	2000	1	2.75E-05	2.20E-02	-0.784	12	0.6	LOC122435727	
X:126104001	X	126104001	126105000	1000	1	1.72E-05	1.85E-02	-0.901	6	0.6		
X:129705001	X	129705001	129707000	2000	1	8.18E-05	3.13E-02	-0.752	11	0.55	LOC122436007;LOC122435205	
X:131964001	X	131964001	131966000	2000	1	4.74E-05	2.66E-02	-0.667	23	1.15	TENM1	
X:135415001	X	135415001	135417000	2000	2	2.95E-06	8.54E-03	-0.996	9	0.45		
X:139249001	X	139249001	139253000	4000	1	8.31E-05	3.13E-02	-0.886	35	0.88		
X:143460001	X	143460001	143461000	1000	1	4.94E-05	2.66E-02	-0.802	9	0.9	LOC122435314	
X:144041001	X	144041001	144042000	1000	1	2.04E-05	2.01E-02	-0.715	0	0	LOC122435077	
X:144170001	X	144170001	144171000	1000	1	1.42E-05	1.76E-02	-0.733	7	0.7		
X:145031001	X	145031001	145032000	1000	1	1.63E-07	3.16E-03	-1.14	4	0.4		
Y:413001	Y	413001	415000	2000	1	5.35E-05	2.71E-02	-1.161	11	0.55		

Supplemental Table S3. Roosevelt Female Elk DMR p<1e-04. DMR name, chromosome number, start nucleotide site, length (bp), p-value, maximum log fold change (LFC), CpG number and density, gene annotation, and gene category.

Supplemental Table S4
Rocky Mountain Male DMR p<1e-04

DMR Name	Chr	Start	Stop	Length	# Sig Win	minP	minFDR	maxLFC	CpG #/ 1Kb	CpG Density/ 100 bp	Gene Annotation	Gene Category
1:8352001	1	8352001	8353000	1000	1	3.21E-05	4.84E-01	-1.475	33	3.3		
6:21556001	6	21556001	21557000	1000	1	4.53E-05	5.02E-01	1.716	13	1.3	LOC122443251;LOC122444185	
7:31329001	7	31329001	31330000	1000	1	3.09E-05	4.84E-01	1.683	11	1.1	TBC1D5	Signaling
13:27626001	13	27626001	27627000	1000	1	6.80E-05	5.77E-01	-1.305	27	2.7	FHAD1	
14:66992001	14	66992001	66998000	6000	1	3.34E-05	4.84E-01	2.266	146	2.43		
14:67000001	14	67000001	67013000	13000	4	9.40E-06	4.84E-01	2.7	270	2.08		
15:71008001	15	71008001	71009000	1000	1	9.45E-05	6.68E-01	-1.09	38	3.8		
17:48851001	17	48851001	48853000	2000	1	3.43E-05	4.84E-01	2.208	19	0.95	NTRK3	Receptor
27:51109001	27	51109001	51111000	2000	1	6.11E-05	5.75E-01	-2.188	35	1.75		

Supplemental Table S4. Rocky Mountain Male Elk DMR p<1e-04. DMR name, chromosome number, start nucleotide site, length (bp), p-value, maximum log fold change (LFC), CpG number and density, gene annotation, and gene category.

Supplemental Table S5
Roosevelt Male DMR Table p<1e-04

DMR Name	Chr	Start	Stop	Length	# Sig Win	minP	minFDR	CpG #/ 1Kb	CpG Density/ 100 bp	CpG Density	Gene Annotation	Gene Category
5:78321001	5	78321001	78322000	1000	1	3.79E-05	4.20E-01	-1.087	24	2.4		
6:4750001	6	4750001	4751000	1000	1	4.23E-05	4.20E-01	1.214	16	1.6		
8:11801001	8	11801001	11802000	1000	1	3.78E-08	3.89E-03	-3.722	21	2.1	LOC122445883	
14:69599001	14	69599001	69607000	8000	2	4.92E-05	4.20E-01	1.871	214	2.67		
14:69608001	14	69608001	69620000	12000	6	2.74E-06	1.41E-01	2.175	308	2.57		
14:69661001	14	69661001	69668000	7000	1	7.82E-05	5.03E-01	1.477	173	2.47		
14:70241001	14	70241001	70245000	4000	1	8.43E-05	5.06E-01	1.296	122	3.05		
14:70246001	14	70246001	70257000	11000	1	5.44E-05	4.20E-01	1.468	222	2.02		
26:11608001	26	11608001	11613000	5000	2	1.29E-05	2.22E-01	-1.378	199	3.98		
26:11821001	26	11821001	11823000	2000	1	5.71E-05	4.20E-01	-1.11	75	3.75		
26:13463001	26	13463001	13466000	3000	1	6.34E-05	4.35E-01	-1.031	126	4.2		

Supplemental Table S5. Roosevelt Male Elk DMR Table p<1e-04. DMR name, chromosome number, start nucleotide site, length (bp), p-value, maximum log fold change (LFC), CpG number and density, gene annotation, and gene category.

Supplemental Table S6
DMR Overlaps Rocky Mountain and Roosevelt Female DMR p<1e-04

DMR Name	Chr	Start	Stop	Length	# Sig Win	minP	minFDR	maxLFC	CpG #/ 1Kb	CpG Density/ 100 bp	Gene Annotation	Gene Category
4:24930001	4	24930001	24931000	1000	1	5.43E-05	2.07E-02	-0.904	5	0.5		
9:63404001	9	63404001	63406000	2000	1	2.74E-05	1.65E-02	-1.353	13	0.65		
14:68001001	14	68001001	68008000	7000	4	6.36E-08	1.25E-03	-2.058	22	0.31	LOC122453182	
20:22827001	20	22827001	22829000	2000	1	6.75E-05	2.32E-02	-1.247	15	0.75	LOC122422607	
25:30458001	25	30458001	30459000	1000	1	2.39E-05	1.54E-02	-0.933	9	0.9	LOC122427759	
28:31388001	28	31388001	31391000	3000	1	9.38E-05	2.66E-02	-1.045	29	0.97	LOC122429464	
X:97589001	X	97589001	97602000	13000	1	3.46E-05	1.72E-02	-1.198	88	0.68	LOC122435661;LOC122435662	
X:99617001	X	99617001	99620000	3000	2	5.13E-05	2.03E-02	-1.284	20	0.67	LOC122435824;LOC122435672;LOC122435863	

Supplemental Table S6. Overlaps between Rocky Mountain and Roosevelt Female Elk DMRs at p<1e-04. DMR name, chromosome number, start nucleotide site, length (bp), p-value, maximum log fold change (LFC), CpG number and density, gene annotation, and gene category.

Supplemental Table S7
DMR Associated Genes and Names

Gene Symbol	Gene Name
ABAT	4-aminobutyrate aminotransferase
ABCB1	ATP-binding cassette, sub-family B (MDR/TAP), member 1
ACACA	acetyl-CoA carboxylase alpha
ACVR1	activin A receptor, type I
ADIPOQ	adiponectin, C1Q and collagen domain containing
ADK	adenosine kinase
ADM2	adrenomedullin 2
AKR1C1	aldo-keto reductase family 1, member C2
AKT2	AKT serine/threonine kinase 2
ANGPTL6	angiopoietin-like 6
B4GALNT1	beta-1,4-N-acetyl-galactosaminyl transferase 1
BID	BH3 interacting domain death agonist
BRSK2	BR serine/threonine kinase 2
BUD31	BUD31 homolog
CACNA1H	calcium channel, voltage-dependent, T type, alpha 1H subunit
CACNA2D3	calcium channel, voltage-dependent, alpha 2/delta subunit 3
CARD9	caspase recruitment domain family, member 9
CD44	CD44 molecule
CDH2	cadherin 2
CEL	carboxyl ester lipase
CFLAR	CASP8 and FADD-like apoptosis regulator
CHRNA2	cholinergic receptor, nicotinic, alpha 2 (neuronal)
CHRNA5	cholinergic receptor, nicotinic, alpha 5 (neuronal)
CHRNB2	cholinergic receptor, nicotinic, beta 2 (neuronal)
CNTN5	contactin 5
CNTNAP2	contactin associated protein-like 2
COL11A2	collagen type XI alpha 2 chain
COMP	cartilage oligomeric matrix protein
CPSF4	cleavage and polyadenylation specific factor 4, 30kDa
CRTC1	CREB regulated transcription coactivator 1
CTBP1	C-terminal binding protein 1
CTBP1	C-terminal binding protein 1
DAB2	disabled homolog 2, mitogen-responsive phosphoprotein
DAGLA	diacylglycerol lipase, alpha
DCC	deleted in colorectal carcinoma
DDX4	DEAD (Asp-Glu-Ala-Asp) box polypeptide 4
DMD	dystrophin
DNAH7	dynein, axonemal, heavy chain 7
EHD2	EH-domain containing 2
EHMT1	euchromatic histone-lysine N-methyltransferase 1
EPHB1	EPH receptor B1
EPHB2	EPH receptor B2
F2	coagulation factor II, thrombin
F2R	coagulation factor II (thrombin) receptor
FFAR4	free fatty acid receptor 4
FRMD4A	FERM domain containing 4A

FSIP1	fibrous sheath interacting protein 1
GALNT2	UDP-N-acetyl-alpha-D-galactosamine:polypeptide N-acetylgalactosaminyltransferase 2 (GalNAc-T2)
GAP43	growth associated protein 43
GFER	growth factor, augmenter of liver regeneration
GRID2	glutamate ionotropic receptor delta type subunit 2
GRM3	glutamate receptor, metabotropic 3
GRPR	gastrin releasing peptide receptor
HOMER1	homer homolog 1 (Drosophila)
HOXA5	homeobox A5
HOXC8	homeobox C8
IGF2	insulin-like growth factor 2 (somatomedin A)
IL2RB	interleukin 2 receptor, beta chain
INSIG2	insulin induced gene 2
IL16	interleukin 16
INS	insulin 1
KAZALD1	Kazal-type serine peptidase inhibitor domain 1
KCNH5	potassium voltage-gated channel, subfamily H (eag-related), member 5
KCNMB3	potassium large conductance calcium-activated channel, subfamily M beta member 3
KCNQ1	potassium voltage-gated channel, KQT-like subfamily, member 1
KLK3	kallikrein-related peptidase 3
KCNQ1	potassium voltage-gated channel, KQT-like subfamily, member 1
LRP1	low density lipoprotein receptor-related protein 1
MAGEB2	MAGE Family Member B2
MAGEB4	MAGE Family Member B4
MED15	mediator complex subunit 15
METRNL	meteorin, glial cell differentiation regulator-like
MUC2	mucin 2, oligomeric mucus/gel-forming
NAV1	neuron navigator 1
NAXD	NAD(P)HX dehydratase
NECTIN2	nectin cell adhesion molecule 2
NFATC1	nuclear factor of activated T-cells, cytoplasmic, calcineurin-dependent 1
NFE2	nuclear factor, erythroid 2
NLGN1	neuroligin 1
NOD1	nucleotide-binding oligomerization domain containing 1
NOS2	nitric oxide synthase 2, inducible
NOTCH1	notch receptor 1
OSPB	oxysterol binding protein
PARN	poly(A)-specific ribonuclease
PDAP1	PDGFA associated protein 1
PICK1	protein interacting with PRKCA 1
PIGU	phosphatidylinositol glycan anchor biosynthesis, class U
PIP5K1C	phosphatidylinositol-4-phosphate 5-kinase, type I, gamma
PLA2G6	phospholipase A2 group VI
PLXNB2	plexin B2
PPAN	peter pan homolog
PREP	prolyl endopeptidase
PRKD1	protein kinase D1
PSMA4	proteasome (prosome, macropain) subunit, alpha type, 4
PTGER3	prostaglandin E receptor 3 (subtype EP3)
PVR	PVR cell adhesion molecule
PXN	paxillin

RAC2	ras-related C3 botulinum toxin substrate 2 (rho family, small GTP binding protein Rac2)
RACK1	Receptor of activated protein kinase C 1
RELA	RELA proto-oncogene, NF-kB subunit
RET	ret proto-oncogene
RORC	RAR-related orphan receptor C
RPL3	ribosomal protein L3
RPLP0	ribosomal protein lateral stalk subunit P0
RPS6KA6	ribosomal protein S6 kinase, 90kDa, polypeptide 6
SCN5A	sodium channel, voltage-gated, type V, alpha subunit
SEMA3D	semaphorin 3D
SHANK3	SH3 and multiple ankyrin repeat domains 3
SLC6A1	solute carrier family 6 (neurotransmitter transporter), member 1
SLC6A2	solute carrier family 6 member 2
TANGO2	transport and golgi organization 2 homolog (Drosophila)
TBC1D9	TBC1 domain family, member 9 (with GRAM domain)
TGFB1	transforming growth factor, beta 1
TH	tyrosine hydroxylase
TOMM20	translocase of outer mitochondrial membrane 20 homolog
TSC2	tuberous sclerosis 2
TSPAN8	tetraspanin 8
TNNT2	troponin T type 2 (cardiac)
USP25	ubiquitin specific peptidase 25
WDFY3	WD repeat and FYVE domain containing 3
WIPF1	WAS/WASL interacting protein family, member 1

Supplemental Table S7. DMR Associated Genes and Names. Gene symbol and name listed.
